# Boosting Antioxidant Self-defenses by Grafting Astrocytes Rejuvenates the Aged Microenvironment and Mitigates Nigrostriatal Toxicity in Parkinsonian Brain *via* an *Nrf2-Driven Wnt/β-Catenin* Prosurvival Axis

**DOI:** 10.3389/fnagi.2020.00024

**Published:** 2020-03-12

**Authors:** Maria Francesca Serapide, Francesca L’Episcopo, Cataldo Tirolo, Nunzio Testa, Salvatore Caniglia, Carmela Giachino, Bianca Marchetti

**Affiliations:** ^1^Pharmacology Section, Department of Biomedical and Biotechnological Sciences, Medical School, University of Catania, Catania, Italy; ^2^Section of Neuropharmacology, OASI Research Institute-IRCCS, Troina, Italy

**Keywords:** Parkinson’s disease, aging, astrocyte–neuron crosstalk, neuroinflammation, dopaminergic neurons, neuroprotection

## Abstract

Astrocyte (As) bidirectional dialog with neurons plays a fundamental role in major homeostatic brain functions, particularly providing metabolic support and antioxidant self-defense against reactive oxygen (ROS) and nitrogen species (RNS) *via* the activation of *NF-E2-related factor 2* (*Nrf2*), a master regulator of oxidative stress. Disruption of As–neuron crosstalk is chiefly involved in neuronal degeneration observed in Parkinson’s disease (PD), the most common movement disorder characterized by the selective degeneration of dopaminergic (DAergic) cell bodies of the substantia nigra (SN) pars compacta (SNpc). Ventral midbrain (VM)-As are recognized to exert an important role in DAergic neuroprotection *via* the expression of a variety of factors, including wingless-related MMTV integration site 1 (*Wnt1*), a principal player in DAergic neurogenesis. However, whether As, by themselves, might fulfill the role of chief players in DAergic neurorestoration of aged PD mice is presently unresolved. Here, we used primary postnatal mouse VM-As as a graft source for unilateral transplantation above the SN of aged 1-methyl-4-phenyl-1,2,3,6-tetrahydropyridine (MPTP) mice after the onset of motor symptoms. Spatio-temporal analyses documented that the engrafted cells promoted: (i) a time-dependent nigrostriatal rescue along with increased high-affinity synaptosomal DA uptake and counteraction of motor deficit, as compared to mock-grafted counterparts; and (ii) a restoration of the impaired microenvironment *via* upregulation of As antioxidant self-defense through the activation of *Nrf2/Wnt/β-catenin* signaling, suggesting that grafting As has the potential to switch the SN neurorescue-unfriendly environment to a beneficial antioxidant/anti-inflammatory prosurvival milieu. These findings highlight As-derived factors/mechanisms as the crucial key for successful therapeutic outcomes in PD.

## Introduction

Astrocyte (As) bidirectional dialog with neurons plays a fundamental role in major homeostatic brain functions. Besides their physical and metabolic support to neurons, As regulate central nervous system (CNS) synaptogenesis, promote neuronal development and plasticity, guide axon pathfinding, modulate the blood–brain barrier, and contribute to neuroprotection *via* the production of different growth and neurotrophic factors, antioxidant and anti-inflammatory molecules, through a concerted crosstalk with neurons (Marchetti and Abbracchio, 2005; Bélanger and Magistretti, 2009; Sofroniew and Vinters, 2010; Molofsky et al., 2012; Sun and Jacobs, 2012). Notably, As display region-specific properties, as the nature of As-derived factors can vary as a function of the CNS region, the age and sex of the host, and the type of brain lesion/injury (Gallo et al., 1995; Marchetti, 1997; Barkho et al., 2006; Jiao and Chen, 2008; Oberheim et al., 2012). Specifically, As of the ventral midbrain (VM-As) represent a primary source of survival, neurotrophic and neuroprotective molecules for dopaminergic (DAergic) neurons (Engele and Bohn, 1991; Takeshima et al., 1994; Morale et al., 2006; Sandhu et al., 2009; L’Episcopo et al., 2010a), the neuronal cell population that progressively degenerates in Parkinson’s disease (PD).

Here, the selective death of DAergic neurons of the substantia nigra pars compacta (SNpc) and their terminals in the striatum (Str) are responsible for the gradual impairment of motor function leading to the classical motor features of PD (i.e., bradykinesia, rest tremor, rigidity, and postural instability; Schapira et al., 2014; Jankovic, 2019). While the causes and mechanisms are not completely understood, current evidence indicates that a complex interplay between several genes and many environmental factors affecting the regulation of crucial pathways involved in inflammatory glial activation, mitochondrial function, protein misfolding/aggregation, and autophagy contribute to DAergic neuron demise in PD (Marchetti and Abbracchio, 2005; Frank-Cannon et al., 2008; Gao et al., 2011; Lastres-Becker et al., 2012; Cannon and Greenamyre, 2013; Hirsch et al., 2013; Dzamko et al., 2015, 2017; Langston, 2017; Blauwendraat et al., 2019).

Especially, in this context, dopamine (DA) oxidative metabolism represents a vulnerability factor linking both mitochondrial and lysosomal dysfunctions to PD pathogenesis (Hirsch and Hunot, 2009; Johri and Beal, 2012), whereby As play a critical antioxidant self-protective role. Hence, oxidative stress upregulates the expression of *Nuclear factor erythroid 2* like 2 (*NFE2L2/Nrf2*), which translocates to the nucleus and binds to antioxidant responsive elements (AREs; Tebay et al., 2015; Zhang et al., 2017). Besides other activated genes, the antioxidant, anti-inflammatory, and cytoprotective *Heme oxygenase 1* (*HO1*) and superoxide dismutase 1 (SOD1; Chen et al., 2009; Sandhu et al., 2009; Surh et al., 2009; Zhang et al., 2017) likely play an important role in DAergic neuroprotection against oxidative damage (Burbulla et al., 2017; Giguère et al., 2018; Surmeier, 2018; Nguyen et al., 2019).

Notably, aging represents a chief risk factor for PD development, as with advancing age, nigrostriatal DAergic neurons progressively deteriorate (Rodriguez et al., 2015 Wyss-Coray, 2016; Poewe et al., 2017). With age, the “adaptive” or “compensatory” capacity of midbrain DAergic neurons gradually fails, which may contribute to the slow nigrostriatal degeneration of PD (Hornykiewicz, 1993; Bezard and Gross, 1998; Blesa et al., 2017). In fact, aging is associated to a gradual decline in the ability of DAergic neurons to recover upon an insult (Bezard and Gross, 1998; Ho and Blum, 1998; Boger et al., 2010). Aging exacerbates inflammation and oxidative stress, which are crucial hallmarks of PD and 1-methyl-4-phenyl-1,2,3,6-tetrahydropyridine (MPTP)–induced PD (Di Monte and Langston, 1995; Langston, 2017).

In fact, microglial cells show age-dependent and region-specific changes in morphology such as structural deterioration or dystrophy, decreased expression of growth/neurotrophic factors, and an impaired phagocytic activity in the face of increased marker expression and upregulation of pro-inflammatory molecules (see Niraula et al., 2017, and references herein), all of which are associated to a gradual loss of As and microglia neuroprotective capacity. Reportedly, microglia switch to a so-called “primed” status, endowed with a strong neurotoxic, pro-inflammatory M1 phenotype with harmful consequences for As–neuron interactions (Miller and Streit, 2007; Liddelow et al., 2017; Rosciszewski et al., 2018, 2019) and DAergic neuron survival upon injury (L’Episcopo et al., 2011a,b,c, 2018a,b).

Notably, genetics, environmental toxicity, and particularly “inflammaging” differentially affect the nature, quality, and outcome of As–neuron crosstalk, directing to either neurodegeneration/repair (L’Episcopo et al., 2018a,b). Hence, the neuroprotective functions of VM-As are impaired, including their ability to mount a self-protective neurorepair strategy thanks to the expression of several pro-neurogenic, neurotrophic, and antioxidant molecules (Marchetti et al., 2013).

Besides others, we uncovered the *Wnt/β-catenin* signaling pathway, a chief player in neurodevelopmental processes (Salinas, 2012; Arenas, 2014; Joksimovic and Awatramani, 2014; Wurst and Prakash, 2014; Brodski et al., 2019; Marchetti et al., 2020), as a crucial signaling system involved in the physiopathology of nigrostriatal DAergic neurons (see Marchetti, 2018, for extensive review). The hallmark of the Wnt/β-catenin pathway after binding the Wnt’s receptors, Frizleds (Fzds), is the cytoplasmic accumulation of β-catenin and its nuclear translocation, finally activating the transcription of Wnt target genes involved in DAergic neurogenesis and neuroprotection (Marchetti, 2018). Notably, VM-As express region-specific transcription factors including Wnt glycoproteins (Marchetti et al., 2013). Especially, wingless-related MMTV integration site 1 (*Wnt1*) is critically involved in DAergic neuroprotection against several neurotoxic and inflammatory insults (Marchetti and Pluchino, 2013).

Of specific mention, with age, Wnt signaling becomes dysfunctional, with potential consequences for neuron–glia crosstalk, DAergic neuron plasticity, and repair (Marchetti, 2018). Notably, deregulation of Wnt signaling has been reported in major neurodegenerative diseases including PD (Berwick and Harvey, 2012; Galli et al., 2014; Harvey and Marchetti, 2014; Marchetti, 2018; Tapia-Rojas and Inestrosa, 2018; Palomer et al., 2019). Additionally, an increasing number of reports corroborate a Wnt connection for neuron survival and regeneration (Singh et al., 2016; Wang et al., 2017; Marchetti et al., 2020).

To date, there are no effective treatments that can stop or reverse the neurodegeneration process in PD, and current treatments rely on DAergic drugs, including levodopa (L-DOPA) and DAergic agonists, which only temporarily alleviate motor symptoms (Olanow and Schapira, 2013; Obeso et al., 2017; Olanow, 2019). Thus, different lines of research are being pursued to develop novel therapeutic regimens for PD, including cell therapies, aimed at protecting or enhancing the intrinsic regenerative potential of DAergic neurons.

Hence, an increasing number of studies focus on the ability of neural stem progenitor cells (NSCs) harvested from the adult brain to engraft in the injured brain of experimental neurodegenerative diseases and exert positive effects (Yasuhara et al., 2006; Redmond et al., 2007) promoting local trophic support and immune modulation, thus synergizing with the restorative responses of the endogenous NSC population (Madhavan et al., 2009; Zuo et al., 2015; Bacigaluppi et al., 2016). Recently, we performed a carefully constructed time-course analysis of the degenerative changes occurring at the nigrostriatal level of aged male mice upon exposure to MPTP and studied the effects of the unilateral transplantation of syngeneic somatic NSCs within the SNpc. In this aged mouse model, the compensatory DAergic mechanisms are lost, and MPTP induces a long-lasting nigrostriatal degeneration with no repair (Collier et al., 2007).

Interestingly, we found that grafted adult NSCs mostly differentiated into reactive As activating intrinsic cues instructing endogenous As to incite DAergic neuroprotection/neurorestoration, thus efficiently counteracting neurotoxin-induced long-lasting DA degeneration (L’Episcopo et al., 2018b).

Based on this background, VM-As appear uniquely positioned to drive neuroprotective and regenerative programs in PD, and recent preclinical studies used co-transplantation of As to promote the regenerative effects of co-transplanted stem cells in rodent PD models (Yang et al., 2014; Song et al., 2018). However, how the aged brain responds to pharmacological or cellular therapeutical interventions is not well documented. In particular, whether transplantation of VM-As by themselves might fulfill the role of chief players in DAergic neurorestoration of aged PD mice is presently unresolved.

Here, we combined different recognized environmental risk factors for human PD, i.e., aging, male gender, inflammation, and exposure to MPTP (Langston, 2017), to explore the capacity of As grafting to mitigate the harmful SN microenvironment and DAergic toxicity. To this end, we used primary mouse postnatal [postnatal days 2–3 (P2–3)] VM-As as a graft source for unilateral transplantation above the SN of middle-aged mice treated with MPTP, after the onset of motor symptoms. We herein report that the engrafted VM-As survived, expressed As markers, and promoted a remarkable reduction of DAergic neuron loss within the MPTP-lesioned SN associated to striatal DAergic reinnervation and functionality. Gene expression analyses coupled to immunofluorescence and functional data *in vivo, ex vivo*, and *in vitro*, suggest the ability of AS grafts to increase Nrf2-antioxidant self-defense, to mitigate MPTP-induced oxidative stress and inflammation, thus switching the harmful As–neuron crosstalk, *via* activation of an *Nrf2/Wnt/β-catenin* prosurvival axis.

## Materials and Methods

### Mice and Treatments

Middle-aged (9- to 11-month-old) male C57BL/J (Charles River, Calco, Italy) mice were maintained under standard laboratory conditions. All surgeries were performed under anesthesia. The mice received *n* = 4 intraperitoneal (i.p.) injections of vehicle (saline) or MPTP-HCl (Sigma-Adrich, St. Louis, MO, USA) dissolved in saline, 3 h apart during 1 day, at a dose of 12 mg/kg^−1^ free base, according to titration studies that produced long-lasting depletion of DA end points with no recovery in both Str and SNpc of aged mice without causing toxicity (L’Episcopo et al., 2013). MPTP was handled in accordance with the reported guidelines (Jackson-Lewis and Przedborski, 2007). Based on our time-course analysis of the degenerative changes occurring in aging male mice upon exposure to MPTP (L’Episcopo et al., 2013, 2018b), a window of 7 days post-MPTP was selected for transplantation of VM-As (Supplementary Figure S1).

### Experimental Design

Middle-aged male mice exhibiting a long-lasting nigrostriatal DAergic toxicity with no recovery upon MPTP treatment (L’Episcopo et al., 2018b) were used to study the neuroprotective/neurorescue effects of unilateral transplantation of As derived from the ventral midbrain (tVM-As), above the SN (Supplementary Figure S1). We designed in *vivo*, *ex vivo*, and *in vitro* experiments. The studies included the SN and the Str and were performed both in basal condition and at different time points (tps) after MPTP ± VM-As/mock transplants, covering 1–5 weeks post-MPTP. The quantification of the different parameters studied included immunohistochemical, neurochemical, gene expression, and behavioral analyses (see Supplementary Figure S1). Based on our gene profiling analysis of the MPTP response in young and aged mice (L’Episcopo et al., 2011b, 2013, 2018b), this study focused on inflammation and oxidative stress, and Wnt signaling genes that are specifically altered in aging mice (see Marchetti, 2018). The microglial response was studied using quantitative immunohistochemistry, and at both gene and protein levels. For the As response, both *ex vivo* and *in vitro* experiments were carried out to address molecular and functional changes according to: (i) the different experimental groups; (ii) the different pharmacological challenges; and (iii) the different As–neuron coculture paradigms established with primary mesencephalic neurons, according to our previous studies (L’Episcopo et al., 2011b, 2014a, 2018a,b).

### Ventral Midbrain As Cultures

**Figure 1:**
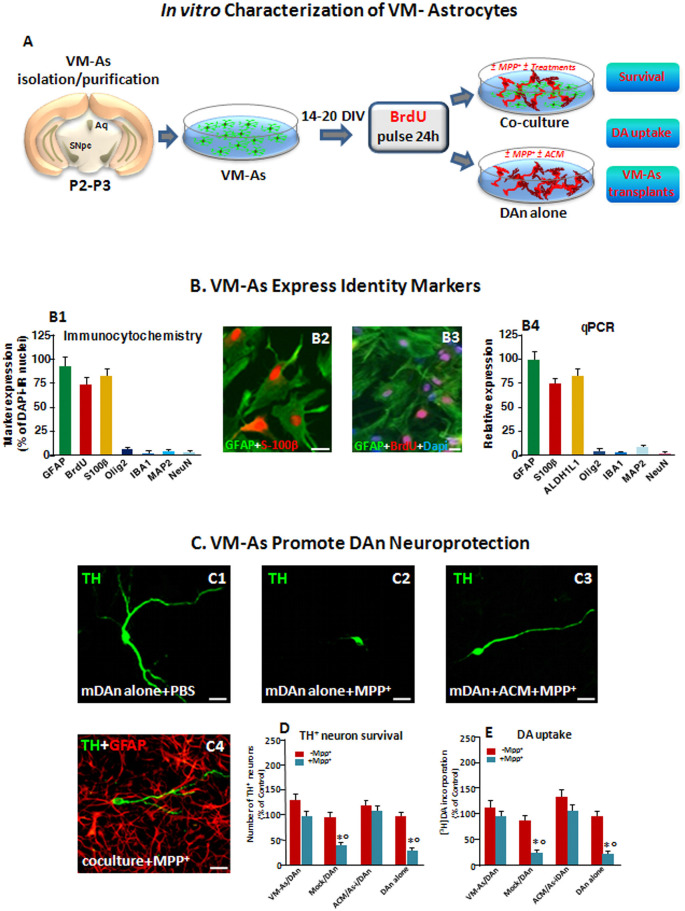
Identity markers and neuroprotective properties of primary ventral midbrain astrocytes (VM-As). **(A)** Scheme of VM-As isolation and purification and direct (coculture) or indirect (As-conditioned medium, ACM) culture paradigms with purified primary mesencephalic dopaminergic neurons (DAn). VM-As pulsed with bromodeoxyuridine (BrdU) were used as a graft source for transplantation. **(B)** Expression of As markers estimated by immunocytochemical (**B1–B3**; *n* = 3 independent experiments) and quantitative real-time (qPCR, **B4**) analyses (*n* = 3 replicates). Quantification of proliferation, glial or neural differentiation markers (mean ± SEM) expressed as percentage of immunoreactive (IR) cells over total glial fibrillary acidic protein–positive (GFAP^+^)/Dapi^+^ cells **(B1–B3)**, and qPCR analysis **(B4)** supported the astrocytic identity of the cultures, revealed by co-expression with S100β (**B1,B2**, in red) and ALDH1L1 **(B4)** and the poor expression of IBA1, Olig2, and MAP2 or NeuN, at both gene and protein levels **(B1,B4)**. Bars: 25 μm. When pulsed with the nucleotide analog, BrdU, a large proportion of GFAP^+^ cells were co-stained after 24 h (B3). **(C–E)** VM-As promote DAn neuroprotection against MPP^+^. **(C1–C4)** Confocal images of tyrosine hydroxylase–positive (TH^+^; green) neurons cultured alone in the absence **(C1)** or presence of MPP^+^ without **(C2)** or in the presence of ACM, or in coculture with VM-As **(C4)**. *Bars*: 25 μm. **(D)** Quantification of TH^+^ neuron survival at 10 days *in vitro* (DIV) in the different culture paradigms (direct or indirect coculture between VM-As or mock with purified DAn vs. DAn alone) and DA uptake levels measured by [^3^H]DA incorporation (**E**; *n* = 3 independent experiments). Comparable results were obtained in the indirect coculture paradigms (DAn exposure to ACM or to VM-As inserts, As-), and values were pooled together. *p ≤ 0.01 vs. -MPP^+^ within the same experimental group; °*p* ≤ 0.01 vs. VM-As groups (direct and indirect coculture) by ANOVA followed by Newman–Keuls test.

**Figure 2:**
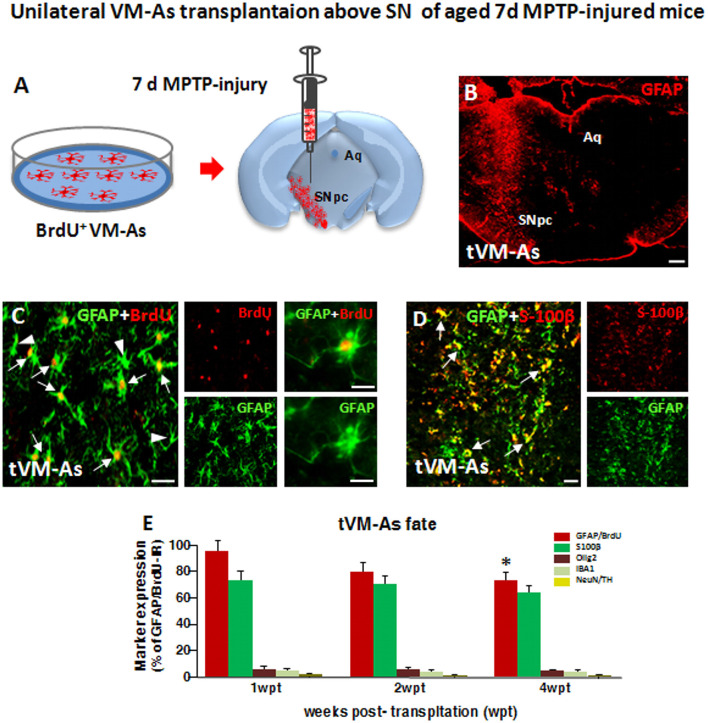
Grafted VM-As survive, express identity markers, and integrate into the aged 1-methyl-4-phenyl-1,2,3,6-tetrahydropyridine (MPTP)-lesioned host substantia nigra pars compacta (SNpc). **(A)** Purified VM-As pulsed with BrdU were unilaterally transplanted above the SNpc, and analyses were carried out 1–4 weeks post-implant (wpt). **(B)** Confocal microscopic image of a coronal midbrain section at the level of the SNpc showing GFAP^+^ As injection. Scale bar: 600 μm. **(C,D)** Grafted GFAP^+^ astrocytes expressing BrdU (**C**, arrows) and BrdU^−^ (**C**, arrowhead) cells and S100β **(D)** are shown. *Scale bars*: **(C)**, 50 μm; **(C)** magnification, 20 μm; **(D)**, 50 μm. **(E)** Relative quantification of transplanted BrdU^+^GFAP^+^ astrocytes at the SN level. Data (mean± SEM, *n* = 6 brains/time point, tp) are percentage of BrdU^+^GFAP^+^ cells over 1 wpt (100%) and expression of glial or neural differentiation markers by grafted VM-As at 1–3 wpt. Time-course analyses indicated that more than 60–70% of the engrafted BrdU^+^GFAP^+^ cells expressed S100β, whereas only 2–3% of tVM-astros were IR for the oligodendroglial cell marker Olig2^+^ and the microglial marker IBA1, and only occasionally did BrdU^+^/GFAP^+^ cells colocalize with neuronal (NeuN) or DAergic (TH) markers. By 4 wpt, an almost 25–30% decline of BrdU^+^/GFAP^+^/S100β^+^ astrocytes was observed within the lesioned SNpc. Data (mean ± SEM) expressed as percentage of IR cells over total BrdU^+^GFAP^+^ cells. *p ≤ 0.05 vs. 1 wpt by ANOVA followed by Newman–Keuls test.

Primary astroglial cell cultures were obtained from mouse VM at P2–3 according to Booher and Sensenbrenner (1972), with slight modifications, as described in full detail (Gallo et al., 2000a; Gennuso et al., 2004). The cultures were allowed to grow and differentiate until they reached confluency, at which time (15–20 days *in vitro*, DIV) the loosely adherent microglial cells were separated by shaking for 2 h at 37°C and 190 rpm. The attached cells were then washed with sterile phosphate buffered saline (PBS) and incubated for 1–2 h at 37°C, at 5% CO^2^, before overnight shaking at 37°C and 210 rpm. The supernatant media containing oligodendrocyte precursors and other cell types were discarded. The glial [more than 95% of the cells were glial fibrillary acidic protein (GFAP)–immunoreactive (IR) As] monolayers were then rinsed with sterile PBS and pulsed with the nucleotide analog bromodeoxyuridine (BrdU, 5 μM) 24 h before transplantation. Part of the cultures were replated at a final density of 0.4–0.6 × 10^5^ cells/cm^2^ in poly-D-lysine (10 μg/ml)–coated 6-, 12- or 24-well plates, or in insert membranes (0.4 μm polyethylene terephthalate) for direct or indirect coculture (BD Biosciences) with primary mesencephalic neurons, and processed as described for RT-PCR or immunocytochemistry (Figures 1, 2).

### Transplantation of VM-As

Upon verification of the purity, proliferation, and neuroprotective potential, VM-As, tagged *in vitro* with BrdU, were then transplanted at different concentrations in pilot experiments conducted for optimization of cell number (50–200 × 10^3^ VM-As; *n* = 6 mice/experimental group) and timing of transplantation after MPTP (1, 7, or 21 days post-MPTP, *n* = 6 mice/tp). The dose of 150 × 10^3^ VM-As and 7-day post-MPTP interval were selected, as a higher number of VM-As and TH^+^ neurons were recovered by 1 week post-implant (wpt). On the day of transplantation (i.e., day 7 post-MPTP), MPTP-injected mice exhibiting a significant motor deficit were randomly (Supplementary Figure S1) assigned to MPTP + PBS, MPTP + VM-As grafts, or MPTP + mock [VM dead cells (VMCs), Madhavan et al., 2009]. Mice were anesthetized with chloral hydrate (600 mg/kg) and positioned in a stereotaxic apparatus. The following stereotaxic coordinates were used: 3.2 posterior to the Bregma, 1.5 mm lateral to the midline, and 3.6 mm ventral to the surface of the dura mater. VM-As or mock (150 × 10^3^) was injected unilaterally above the left SN (over a period of 2 min). The needle was kept in place for 5 min after each infusion before retraction. Saline-injected controls received the same volume of PBS.

### Motor Behavior Analysis With the Rotarod

An accelerating rotarod (five-lane accelerating rotarod; Ugo Basile, Comerio, Italy) was used to measure motor coordination in mice. Mice had to keep their balance on a horizontal rotating rod (diameter, 3 cm) and rotation speed was increased every 30 s by 4 rpm. Five mice were tested at the same time, separated by large disks. A trial started when the mouse was placed on the rotating rod, and it stopped when the mouse fell down or when 5 min were completed. Falling down activated a switch that automatically stopped a timer. On the testing day, each mouse was submitted to five trials with an intertrial interval of 30 min. Mice housed five per cage were acclimated to a 12 h shift in light/dark cycle so that the exercise occurred during the animals’ normal wake period. Saline- and MPTP-treated mice (10/experimental group) were assessed for their rotarod performance on days −7, 1, 7, 14, 21, and 28 after MPTP injection.

### Immunohistochemistry

On the day of sacrifice, mice were anesthetized and transcardially perfused with 0.9% saline, followed by 4% paraformaldehyde in phosphate buffer (pH 7.2 at 4°C); the brains were carefully removed and processed as described in full detail (Morale et al., 2004). Tissues were frozen and stored at −80° C until further analyses. Serial coronal sections (14 μm thick), encompassing the Str (Bregma 1.54 to Bregma −0.46) and the SNpc (Bregma −2.92 to Bregma −3.8 mm) according to Franklin and Paxinos (2007), were collected, mounted on poly-L-lysine–coated slides, and processed as previously described in full detail (L’Episcopo et al., 2011b). The following pre-absorbed primary antibodies were used: rabbit anti-tyrosine hydroxylase (TH, Chemicon International, Temecula, CA, USA), the rate limiting enzyme in DA synthesis; rabbit anti-TH (Peel Freez Biochemicals, Rogers, AR, USA); mouse anti-TH (Boehringer Mannheim Bioc., Philadelphia, PA, USA), rat anti-dopamine transporter (DAT, Chemicon, Int. USA); mouse anti-neuron specific nuclear protein (NeuN, US Biologicals, Swampscott, MA, USA); rabbit anti-GFAP (GFAP, Dako, Cytomation, Denmark), mouse anti-GFAP (Sigma-Adrich, St. Louis, MO, USA) as an As-specific cell marker; goat anti-ionized calcium-binding adapter molecule 1 (IBA1, Novus Biologicals, Littleton, CO, USA) a microglia-specific marker; goat anti-heme oxygenase 1 (anti-Hmox, 1:150, Santa Cruz Biotechnology, Santa Cruz, CA, USA); rabbit polyclonal, anti-inducible nitric oxide synthase (iNOS;1:200; Santa Cruz Biotechnology, Santa Cruz, CA, USA); and rabbit anti-3-nitrotyrosine (3-NT; 1:200; Millipore, Kankakee, IL, USA; see also complete list of Abs in Supplementary Table S1). Nuclei were counterstained with 4′,6-diamidino-2-phenylindole (DAPI) in mounting medium (Vector Laboratories, Burlingame, CA, USA). Visualization of incorporated BrdU requires DNA denaturation performed by incubating the sections in HCl for 30 min at 65°C. After overnight incubation, sections were washed extensively and incubated with fluorochrome (FITC, CY3, CY5)–conjugated species-specific secondary antibodies for immunofluorescent detection. TH immunoreactivity was also detected using biotinylated secondary antibodies (Vector Laboratories, Burlingame, CA, USA) and diaminobenzidine (DAB, Vector Laboratories, Burlingame, CA, USA) as the developing agent as described (L’Episcopo et al., 2011b). Cresyl violet (CV) was used to visualize the Nissl substance.

In all of these protocols, blanks were processed as for experimental samples except that the primary antibodies were replaced with PBS.

### DAergic End Points

Quantitative analysis of DAergic neurons in the SNpc was carried out by serial section analysis of the total number of TH-positive (TH^+^) and NeuN-positive (NeuN^+^) neurons throughout the entire rostro-caudal axis of the SNpc (Franklin and Paxinos, 2007) as previously described (L’Episcopo et al., 2011b). Total numbers of TH- and CV-stained neurons in adjacent tissue sections were estimated in parallel to validate TH^+^ neuron survival, using Abercrombie correction (Abercrombie, 1946).

Striatal TH- and DAT-immunoreactive (IR) fiber staining was assessed in *n* = 3 coronal sections at three levels (Bregma coordinates: +0.5, +0.86, and 1.1 mm, respectively) of the caudate putamen (CPu), in *n* = 6 mice/group/time (Burke et al., 1990). Fluorescence intensity (FI) of TH- and DAT-staining above a fixed threshold used the corpus callosum for background subtraction.

For synaptosomal, high-affinity DA uptake, at the indicated time intervals, mice were sacrificed by cervical dislocation, and the brains rapidly removed and immediately placed on ice-cold saline. The right and left striata were then dissected on an ice-cold plastic dish and processed as described in full detail (L’Episcopo et al., 2011a,b). Synaptosomal, high-affinity DA uptake was assessed in the presence of 10 μM mazindol, according to Morale et al. (2004).

### Glial Cell Counts

Cell counts were obtained for GFAP^+^ As and ameboid IBA1^+^ microglial cells (Kreutzberg, 1996). For SN cell counts, at least three sections were obtained from each animal representing each of the five representative planes from −2.92 mm to −3.8 mm relative to Bregma according to the stereotaxic coordinates of Franklin and Paxinos (2007). GFAP^+^ As and IBA1^+^ microglial cell number per unit of surface area was determined in 8–10 randomly selected fields per section on both sides, the counts averaged for each animal, and the mean number of cells per mm^2^ was estimated. Classification of microglia activation was carried out according to Kreutzberg (1996); as described in Supplementary Table S3, the glial counts were confirmed by two different observers.

### Confocal Laser Scanning Microscopy, Image Analysis, and Quantification of Immunostaining

All the quantifications were performed by investigators blind to treatment conditions. Immunostaining was examined using a Leica LCS-SPE confocal microscope. For FI assessments and colocalizations, midbrain sections were labeled by immunofluorescence, and images were acquired by sequential scanning of 12–16 serial optical sections (Gennuso et al., 2004; L’Episcopo et al., 2011a,b,c, 2013). Three dimensional reconstructions from z-series were used to verify colocalization in the x–y, y–z, and x–z planes. Serial fluorescent images were captured in randomly selected areas, the number of labeled cells per field (*n* = 6–8 fields/section) was manually counted in 4–6 midbrain sections per brain (*n* = 6/treatment group) using Olympus cellSense Dimension software, and cell counts obtained were averaged (mean ± SEM); the percentages of HO1^+^/GFAP^+^ cells out of the total GFAP^+^ cells were estimated in each condition, in ≥100 cells, read from at least four VM sections per brain, in six mice per experimental group, and results expressed as mean ±SEM (Gennuso et al., 2004).

### RNA Extraction, Reverse Transcription, and Real-Time PCR

RNA was extracted from tissues/cell samples, as previously detailed (L’Episcopo et al., 2011a,b). Briefly, after purification using a QIAquick PCR Purification kit (Qiagen), 250 ng of cDNA was used for real-time PCR using pre-developed TaqMan assay reagents (Applied Biosystems). Real-time quantitative PCR was performed using the Step One Detection System (Applied Biosystems) according to the manufacturer’s protocol, using the TaqMan Universal PCR master mix (#4304437). The assay IDs are reported in Supplementary Table S2. For each sample, we designed a duplicate assay. β-actin (Applied Biosystems #4352341E) was selected as the housekeeping gene, according to our previous (L’Episcopo et al., 2011b, 2018b) and present pilot studies indicating that it does not modify its expression between conditions. Quantification of the abundance of target gene expression was determined relative to β-actin with regard to the control group by using the delta C_t_ (2^−ΔΔCt^) comparative method, with the results expressed as arbitrary units (AU). Relative fold changes over saline/PBS or MPTP/PBS are indicated.

### Enriched Neuronal Cultures and Primary Midbrain Astroglial–Neuron Cultures

For *in vitro* establishment of primary mesencephalic neuronal cultures, timed pregnant Sprague–Dawley rats (Charles River Breeding Laboratories, Milan, Italy) were killed in accordance with the Society for Neuroscience guidelines and Italian law. Primary mesencephalic neurons were prepared from the brain on embryonic day 13–14, as detailed (L’Episcopo et al., 2011b). Briefly, mesencephalic tissues were isolated and dissociated with gentle mechanical trituration. Cells were diluted to 1.5 × 10^6^/ml in maintenance medium (MEM supplemented with 10% heat-inactivated FBS, 10% heat-inactivated horse serum, 1 g/L glucose, 2 mM glutamine, 1 mM sodium pyruvate, 100 μM nonessential amino acids, 50 U/ml penicillin, and 50 μg/ml streptomycin) and seeded into 24-well culture plates precoated with poly-D-lysine (20 μg/ml). Plates were maintained at 37°C in a humidified atmosphere of 5% CO_2_ and 95% air. To obtain neuron-enriched cultures, cytosine β-D-arabinofuranoside (Ara-c) was added to a final concentration of 6 μM 36 h after seeding the cells, to suppress glia proliferation (L’Episcopo et al., 2011b). Cultures were changed back to maintenance medium 2 days later and were used for treatment 7 DIV after initial seeding. Neuronal enrichment was verified by immunocytochemistry using GFAP-, TH-, and NeuN-Abs as described. Ara-c treatment reduced glial expression by 95%.

Both purified neuronal cultures and astroglial–neuron cultures at 7 DIV received MPP^+^ (10 μM). The specificity of the As neuroprotective effect was further verified in purified neuronal cultures exposed to astroglial conditioned media (ACM) or to As inserts (As-i, indirect As–neuron coculture). In this experimental paradigm, the inserts containing the As monolayer were added on the top of the purified neurons. These inserts allowed diffusion of factors from the glia monolayer to the mesencephalic neurons and vice versa, without direct contact between cells (Gallo et al., 1995, 2000a,b). DAergic neuron survival was estimated after 24 h, by counting the number of TH^+^ neurons over the DAPI-positive nuclei and TH^+^ neurons expressed as percent (%) of control (-MPTP), and by determination of [^3^H]DA incorporation, which reflects DAergic cell count and functionality. Uptake of [^3^H]DA was performed essentially as previously described, by incubating the cell cultures for 20 min at 37° with 1 μM [^3^H]DA in Krebs-Ringer buffer [16 mM sodium phosphate, 119 mM NaCl, 4.7 mM KCl, 1.8 mM CaCl_2_, 1.2 mM MgSO_4_, 1.3 mM EDTA, and 5.6 mM glucose (pH 7.4)]. Non-specific DA uptake was blocked by mazindol (10 μM). Cells were then collected in 1 N NaOH after washing in ice-cold Krebs-Ringer buffer. Radioactivity was determined by liquid scintillation and specific [^3^H]DA uptake calculated by subtracting the mazindol counts from the wells without the uptake inhibitor (Morale et al., 2004; L’Episcopo et al., 2011a,b,c).

### *Ex vivo* Isolation of As

*Ex vivo* isolation and culture of glial cells from the adult brain were previously detailed (Schwartz and Wilson, 1992; L’Episcopo et al., 2012, 2013). Isolated As from middle-aged mice of the studied groups (m-astro, >95%, GFAP^+^ cells) were counted and plated at a final density of 0.4–0.6 × 10^5^ cells/cm^2^ in poly-D-lysine (10 μg/ml)–coated 6-, 12-, or 24-well plates, and their conditioned media were collected and stored at −70°. Some of the glial cells were exposed to different treatments and processed for qPCR or functional analyses or were used for direct coculture with primary mesencephalic neurons, as detailed in the previous section.

### Mitochondrial Activity With the 3-(4,5-dimethylthiazol-2-yl)-2,5-diphenyltetrazolium Bromide Assay

The colorimetric 3-(4,5-dimethylthiazol-2-yl)-2,5-diphenyltetrazolium bromide (MTT) assay was used to measure mitochondrial functionality in glial cells (Gennuso et al., 2004). Briefly, cells were incubated with 0.25 mg/ml MTT for 3 h at 37°C, and mitochondrial enzyme activity was measured in culture supernatants in a spectrophotometer (Molecular Devices) at 570 nm, with a reference wavelength of 630 nm. Results are expressed as percentage changes of control.

### ROS and RNS

For reactive oxygen species (ROS) measurement, the redox membrane-permeant probe 2′,7′-dichlorofluorescein diacetate (DCFH-DA, 50 μM) was added for 1 h at 37°C, and cells were viewed under the confocal microscope (Gennuso et al., 2004). Measurement of iNOS-derived NO was carried out in cell free supernatant using Griess reagent (Marchetti et al., 2002; L’Episcopo et al., 2011c). To study the effect of inhibition of oxidative and nitrosative stress mediators, the freshly prepared GFAP^+^ cells were cultured in the absence or the presence of the ROS antagonist, apocynin (Apo, 0.5 mM), and the specific iNOS inhibitor, L-Nil [L-N6-(1-iminoethyl)-lysine, 50 μM, Sigma] (Marchetti et al., 2002; Morale et al., 2004), applied after plating and determinations carried out 24–48 h after treatment (Figure 6).

**Figure 3:**
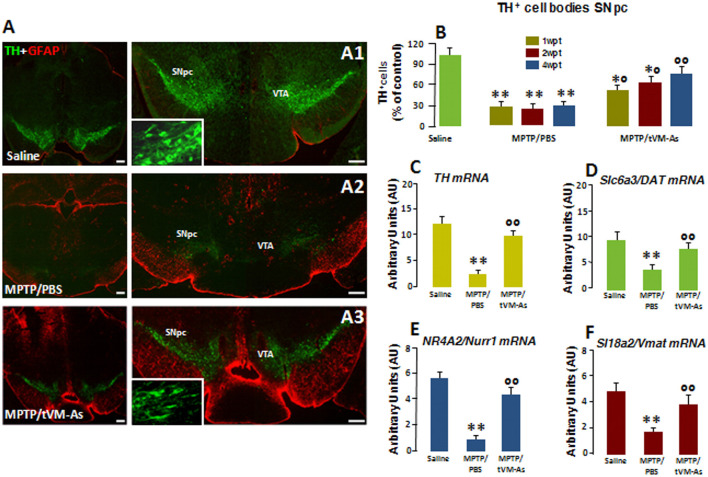
tVM-As promote the rescue of endogenous TH^+^ neurons in the SNpc. **(A1–A3)** Confocal images of the SNpc in middle-aged (9- to 11-month-old) saline-**(A1)**, MPTP/PBS-**(A2)** and aged-matched VM-As–grafted mice **(A3)** at 4 wpt. Scale bars: 600 μm. Magnifications in the *boxed areas*. **(B)** Total number of TH^+^ neurons in the right and left SNpc (mean ± SEM) at 1, 2, and 4 wpt showing that tVM-As grafts increase TH^+^ neuron survival in MPTP mice in a time-dependent fashion. **(C–F)** qRT-PCR in SNpc showing 2- to 3-fold upregulation of typical DAergic transcripts, *TH* **(C)**, *Slc6a3* (*DAT*, **D**), *Nr4A2* (*Nurr1*, **E**), and *Slc18a2* (*Vmat*, **F**) mRNAs of MPTP/tVM-As over 1-methyl-4-phenyl-1,2,3,6-tetrahydropyridine (MPTP)/phosphate buffered saline (PBS). Values (arbitrary units, AU, mean % ± SEM of *n* = 5 samples/experimental group) are expressed as fold changes over control. **p ≤ 0.01. vs. saline/PBS; ^*°^*p* ≤ 0.05. ^°°^*p* ≤ 0.01 vs. MPTP/PBS, at each time interval respectively, by ANOVA followed by *post hoc* Newman–Keuls test.

**Figure 4:**
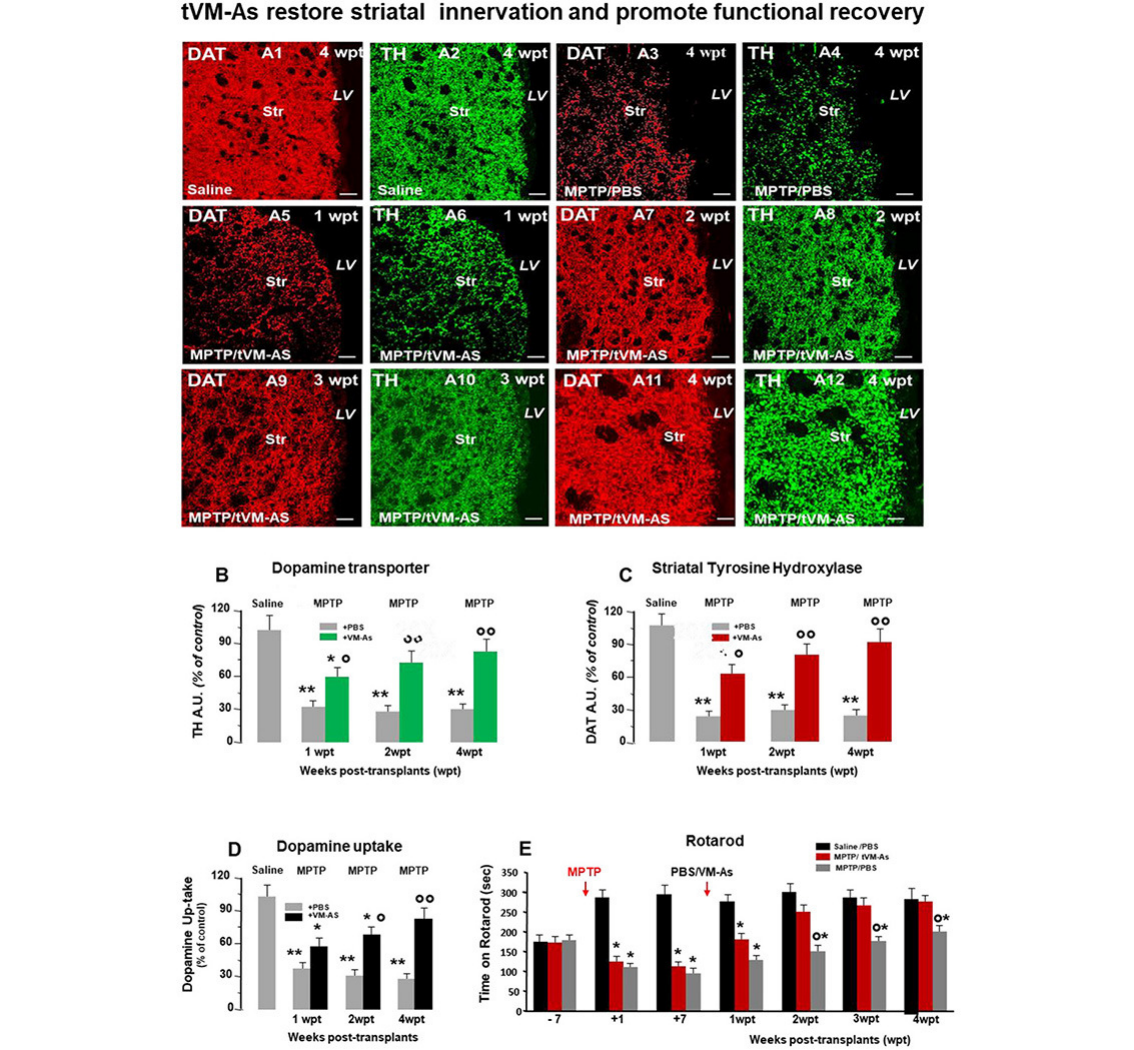
tVM-As grafts counteract MPTP-induced loss of DAergic innervation and synaptosomal dopamine (DA) uptake in the striatum, and revert Parkinson's disease (PD) motor deficits. **(A1–A12)** Representative confocal images of dopamine transporter (DAT, revealed in CY3, red)-fluorescence intensity (FI) and tyrosine hydroxylase (TH, revealed in FITC, green) showing the recognized loss of striatal DAT-IF and TH-IF 4 weeks post MPTP treatment compared to saline **(A1–A4)** as opposed to the ability of tVM-As to counteract MPTP-induced loss of DAT-IF and TH-IF at all time tested **(A5–A12)**. DAT **(B)** and TH-**(C)** immunofluorescent staining measured by image analysis. Scale bars: 50 μm. **(D)** VM-As grafts increase high-affinity striatal (Str) DA uptake assessed by [3H]DA incorporation (mean % SEM). **(E)** Motor performances on rotarod showing recovery from motor impairment in MPTP/tVM-As but not MPTP/PBS mice. ^*^p < 0.05, ^**^p < 0.01 vs. saline/PBS; ^°^*p* < 0.05, ^°°^*p* < 0.01 vs. MPTP/PBS, at each time interval respectively, by ANOVA followed by *post hoc* Newman–Keuls test.

**Figure 5:**
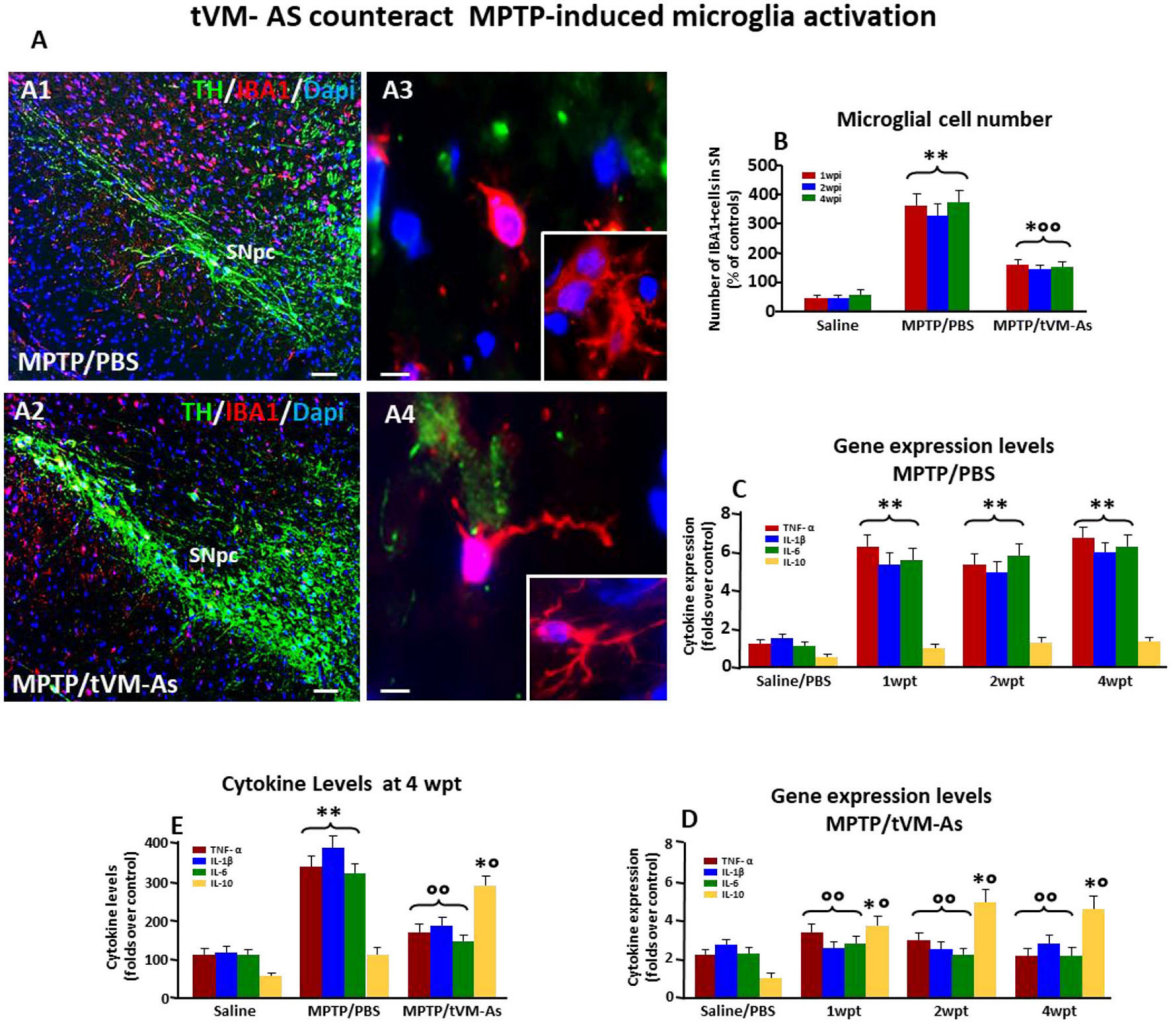
tVM-As downregulate microglial pro-inflammatory phenotype in SNpc. **(A, B)** tVM-As reverse MPTP-induced reactive microglial cells displaying the morphology of activated macrophage-like microglia **(A1,A3)** and the increased IBA1+/Dapi+ microglial cell numbers in midbrain sections at the level of the SNpc **(B)**. Note the ramified microglia in SNpc of tVM-As mice **(A2, A4)**. Scale bars: **(A1, A2)**, 100 μm; **(A3, A4)**, 25 μm. **(C, D)** SNpc tissues were processed for gene expression analyses of mRNA species using qRT-PCR. Values (AU, mean % SEM of *n* = 5 samples/experimental group) are expressed as fold changes. In MPTP/PBS, inflammatory (TNF-α, IL1-β, IL-6) mRNAs are upregulated by about 5- to 6-fold (*p* < 0.01) over saline-injected controls **(C)**, whereas the anti-inflammatory cytokine IL-10 is not affected. Transplantation of VM-As in MPTP mice induced a significant (*p* < 0.01) downregulation of pro-inflammatory markers at all tps but increased IL-10 expression *vs*. MPTP/PBS **(D)**. **(E)** Evaluation of IL-1β, TNF-α, IL-6, and IL-10 at a protein level, as determined by enzyme-linked immunosorbent assay (ELISA) in homogenate tissue samples (mean % SEM of *n* = 5 samples/experimental group), documents the ability of tVM-As to suppress the pro-inflammatory cytokines in the face of a significant increase in the anti-inflammatory cytokine, IL-10, when levels are compared to MPTP/PBS mice. ^*^p < 0.05, ^**^p < 0.01 vs. saline/PBS; ^°°^*p* < 0.01 vs. MPTP/PBS; ^*°^*p* < 0.01 vs. saline/PBS and MPTP/PBS, at each time interval respectively, by ANOVA with post hoc Newman–Keuls.

**Figure 6:**
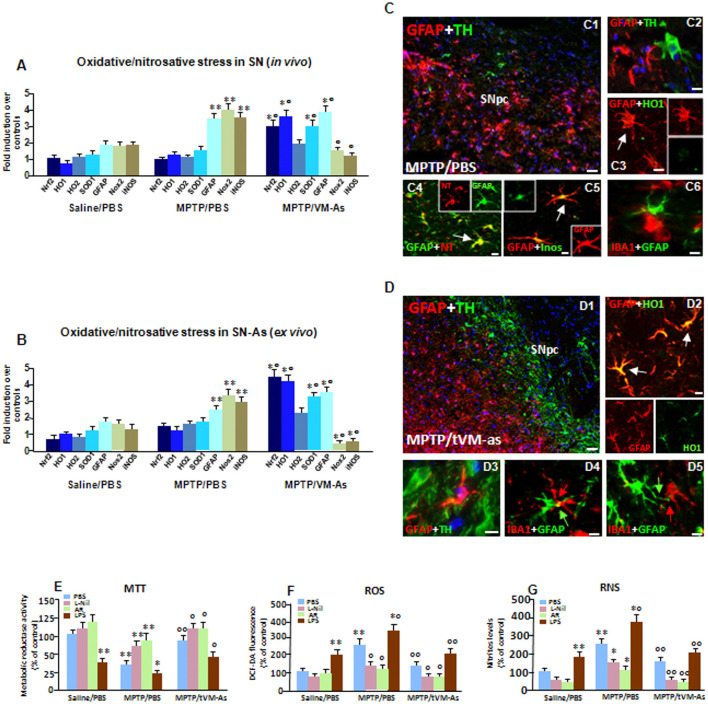
tVM-As upregulate antioxidant self-defense *in vivo and ex vivo*. **(A,B)** Oxidative and nitrosative stress markers were analyzed by quantitative real-time PCR (qPCR) in SNpc tissues derived from MPTP/PBS and MPTP/tVM-As mice, *in vivo* **(A)**, and acutely isolated As, *ex vivo*, from both d groups **(B)**. Values (AU, mean % ± SEM of *n* = 5 samples/experimental group) are expressed as fold changes over control. VM-As grafts upregulated *Nrf2, HO1, and* superoxide dismutase 1 (SOD1), whereas *Nox*2 a*nd* inducible nitric oxide synthase (iNOS) are downregulated vs. MPTP/PBS both in SN tissues **(A)** and As-derived cultures **(B)**. **p ≤ 0.01 vs. saline/PBS; °°*p* ≤ 0.01 vs. MPTP/PBS, within each treatment group, respectively, by ANOVA followed by *post hoc* Newman–Keuls test. **(C,D)** Dual staining with TH (green) and GFAP (red) in midbrain sections from MPTP/PBS **(C1,C2)** and MPTP/tVM-As **(D1–D3)** showing the ability of VM-As grafts to revert MPTP-induced TH neuronal loss. In tVM-As, GFAP^+^ cells extend the long process in close contact, almost embracing TH^+^ neurons (D2, D3, *arrows*), and co-express HO1 **(D4)**. Scale bars: **(C1)**, 100 μm; **(C2–C5)**, 25 μm. By contrast, in MPTP/PBS, GFAP^+^ As (red) do not colocalize with HO (green, **C3**), but express iNOS (green) at high levels **(C5)**. Likewise, the reactive oxygen (ROS) and nitrogen species (RNS) footprint, 3-nitrotyrosine (red), is abundantly expressed in GFAP^+^ (green) cells **(C3)**. Note that in tVM-As–grafted mice, dual staining of GFAP and ionized calcium-binding adapter molecule 1 (IBA1), showing a GFAP^+^ cell in close contact with a ramified IBA1^+^cell phenotype **(D5)**, as opposed to MPTP/PBS, IBA1^+^ cells with a rounded morphology, is seen close to reactive GFAP^+^ As **(C6)**. Scale bars: **(D1)**, 100 μm; **(D2–D4)**, 25 μm. **(E–G)** Accordingly, in astrocyte cultures acutely isolated from MPTP/PBS, the production of ROS measured with the redox membrane-permeant probe 2′,7′-dichlorofluorescein diacetate (DCFH-DA, F) and the generation of iNOS-derived RNS **(G)**, in cell free supernatant, are sharply increased, resulting in a significant inhibition of mitochondrial reductase activity (MTT, **E**). These effects were efficiently counteracted in tVM-As cultures, with the application of the specific iNOS inhibitor, L-N6-(1-iminoethyl)-lysine (L-Nil) **(E–G)**. Activation of Wnt/β-catenin signaling counteracts ROS and RNS production and reverts mitochondrial dysfunction of MPTP/PBS-As **(E–G)**. By contrast, lipopolysaccharide (LPS) application further exacerbated oxidative/nitrosative stress in MPTP/PBS- vs. MPTP/tVM-As (E–G). *p ≤ 0.05, **p ≤ 0.01 vs. saline/PBS; ^*°^*p* ≤ 0.01 vs. saline/PBS and MPTP/PBS; °°*p* ≤ 0.01 vs. MPTP/PBS.

### Enzyme-Linked Immunosorbent Assay

Levels of cytokines were determined in tissue homogenates/cells using enzyme-linked immunosorbent assay (ELISA) kits (DuoSet ELISA Development System; R&D Systems, McKinley Place, MN, USA) following the manufacturer’s protocol (Marchetti et al., 2002; L’Episcopo et al., 2011c).

### Data Analysis

Statistical significance between means ± SEM was analyzed by a two-way analysis of variance (ANOVA). Experimental series performed on different days were compared by the Student–Newman–Keuls *t*-test. A value of *p* < 0.05 was considered to be statistically significant.

## Results

### *In vitro* Characterization of VM-As Displaying Neuroprotective Properties

As a first step of our transplantation protocol, primary VM-As cultures established from P2–3 and cultured as described for 14–20 DIV were processed for qPCR and immunocytochemical analyses to identify astrocytic (GFAP, S100B, ALDHL1) markers and verify the purity of the preparation by testing several non-astrocytic (i.e., microglial IBA1, oligodendrocyte, Olig2, and neuronal MAP2, NeuN) identity markers (Figures 1A,B). As observed, the astrocytic identity was confirmed by As co-expression with S100b, and ALDH1L1, two As-associated genes, whereas IBA1, Olig2, and MAP2 or NeuN were only poorly expressed, at both gene and protein levels (Figure 1B), thus confirming the full differentiation and purity of the VM-astro cultures. Additionally, when pulsed with the nucleotide analog, BrdU, a large proportion of GFAP^+^ cells were co-stained after 24 h.

Our previous studies on neuron-As interactions first reported the region and growth factor specificity of As-derived molecules for neuronal development and growth, for acquisition of the mature neuronal phenotype, as well as for neuroprotection (Gallo et al., 1995; Morale et al., 2006; L’Episcopo et al., 2011a,b). Especially, P2–3 VM-As were shown to promote the differentiation of adult midbrain- but not subventricular zone (SVZ) NSCs into functionally active DAergic neurons, *in vitro* (L’Episcopo et al., 2011b, 2014a). Here the neuroprotective ability of VM-As was further verified in the coculture paradigm, where purified mesencephalic neurons (DAn) were layered on the top of VM-As in both the presence and the absence of the toxic MPTP metabolite, Mpp^+^ (10 μM; Figures 1C–E). In accord with our previous findings (L’Episcopo et al., 2011a,b), in enriched primary mesencephalic neuronal cultures at 7 DIV, MPP^+^ promoted the well-known neuron toxicity as demonstrated by the loss of TH^+^ neurons, DAn atrophy, and inhibition of [^3^DA] incorporation (Figures 1C, 2,D,E). On the other hand, in VM-As–neuron cocultures, MPP^+^ failed to induce DAergic cell death, as reflected by the greater TH^+^ neuronal number and DA uptake levels measured (Figures 1D,E), with the TH^+^ neurons cocultured with VM-As showing longer ramified neurites compared with those without As coculture (Figure 1C2 vs. Figure 1C4). Accordingly, exposure of enriched DAn to either VM-As–conditioned medium (ACM) or VM-As insert paradigms similarly exerted a significant degree of TH^+^ neuron protection against MPP^+^ (Figures 1C3,D,E).

Together, these findings supported the differentiation, proliferative, and neuroprotective properties of our VM-As cultures, *in vitro* (Figures 1A–C).

### Grafted VM-As Survive, Express Identity Markers, and Integrate Into the Aged MPTP-Lesioned Host SNpc

As a second step of our transplantation protocol, we verified the engraftment, proliferation, and distribution of VM-As tagged by incorporation of BrdU, which were unilaterally transplanted 7 days post-MPTP in the SNpc of middle-aged 9- to 11-month-old mice (Figures 2A,B). Spatio-temporal immunohistochemical, neurochemical, molecular, and motor behavioral analyses were carried out 1–3 wpt (Supplementary Figure S1).

By 1 week post-transplant (wpt), tVM-astros showed robust ipsilateral engraftment at all the SN rostro-caudal levels analyzed. Quantitative colocalization analyses in midbrain sections at 1 wpt (1 wpt = 100%) indicated that more than 70% of the engrafted BrdU^+^GFAP^+^ cells expressed S100β, whereas only 3–5% of tVM-astros were IR for the oligodendroglial cell marker Olig2^+^ and the microglial marker IBA1, and we did not observe colocalization of tVM-As with neuronal (NeuN) or DAergic (TH) markers (Figure 2C). Additionally, time-course analyses indicated that by 3 wpt, an almost 25–30% decline of GFAP^+^/BrdU^+^ cells was observed within the lesioned SNpc (Figure 2D).

Together, tVM-As displaying *in vitro* well-recognized neuroprotective features, when transplanted unilaterally in the SNpc of middle-aged mice engrafted within the lesioned SNpc, expressed typical astrocytic, but not neuronal, nor microglial or oligodendroglial, markers and survived up to 4 wpt.

### tVM-As Promote the Rescue of Endogenous TH^+^ Neurons in the SNpc

To investigate the effect of the transplantation of tVM-astros on MPTP-induced neuronal loss in middle-aged mice, the number of DAergic cell bodies in the SNpc was assessed by TH immunoreactivity (Figures 3A1–A3 and Figure 3B). Stereological quantification of TH^+^ and Nissl^+^ neurons in SNpc was performed to validate TH^+^ neuron survival. tVM-astros grafted into MPTP-lesioned mice (MPTP/tVM-As) showed a significant (*p* ≤ 0.05) increase in endogenous TH^+^ immunoreactivity and TH^+^ neuron survival at both early (1 wpt) and later (4 wpt) tps (Figures 3A1–A3 and Figure 3B) vs. control MPTP mice receiving an intranigral injection of PBS (MPTP/PBS; Figures 3A1–A3 and Figure 3B). The observed increase in the number of TH^+^ neurons appears specific for VM-As, since transplantation of a mock cell preparation (VMCs) failed to increase TH^+^ neuron survival (not shown). By 4 wpt, the number of TH^+^ neurons in MPTP/tVM-astro mice became almost comparable with unlesioned (saline/PBS) mice (Figure 3B).

Next, we studied the expression levels of several DAergic mRNA species by applying quantitative real-time polymerase chain reaction (qRT-PCR) in MPTP-injured SNpc tissues. Here, we found that in MPTP/tVM-astro mouse *Th*, the high-affinity DA transporter *Slc6a3* (DAT), the vesicular monoamine transporter *Slc18a2* (VMAT), and the DA-specific transcription factor *Nr4a2* (Nurr1) required for the mature DA phenotype and survival (Kadkhodaei et al., 2009) showed an upregulation (by 2- to 3-fold), vs. MPTP/PBS control mice (Figures 3C–F).

Together, the tVM-As graft had a significant time-dependent rescue effect on endogenous TH neurons, which occurs at tissue, gene, and protein levels. Moreover, this effect was tVM-astro–specific and was not attributable to a direct differentiation of transplanted tVM-astros into TH^+^ neurons but, rather, to a rescue effect on endogenous cells.

### tVM-As Grafts Counteract MPTP-Induced Loss of DAergic Innervation and Synaptosomal DA Uptake in the Str and Revert PD Motor Deficits

We next investigated the ability of tVM-astro grafts to increase the functionality of new TH^+^ neurons, using quantitative confocal laser microscopy on striatal sections, the high-affinity synaptosomal DA uptake levels, and the analysis of motor behavior.

Hence, tVM-As grafts efficiently counteracted the MPTP-induced loss of striatal TH and DAT innervation (Figures 4A1–A12 and Figures 4B–C). By contrast, corresponding levels in control MPTP/PBS mice were found to be significantly lower, compared with both MPTP/tVM-As (*p* ≤ 0.05) and saline/PBS controls (*p* ≤ 0.01) at each tp tested. Measuring the striatal uptake of radiolabeled DA([^3^H]-DA) in presynaptic terminals of middle-aged MPTP mice, we found that MPTP/tVM-As mice showed a significant (*p* ≤ 0.05) recovery of high-affinity striatal synaptosomal DA uptake (vs. MPTP/PBS) by 2 wpt (Figure 4D). This effect in MPTP/tVM-As mice increased over time, reaching values similar to those of saline/PBS controls by 4 wpt. On the contrary, MPTP/PBS mice showed a constant significant (*p* ≤ 0.01) reduction of synaptosomal DA uptake over time, vs. saline/PBS controls.

Behavior analyses confirmed that these structural and functional striatal changes were coupled with a full recovery of motor coordination deficits in MPTP/tVM-As mice, vs. MPTP/PBS controls, which started to be significant (*p* ≤ 0.05) at 2 wpt (Figure 4E).

Together, besides the effects of tVM-As on SNpc-DA neuronal cell bodies, tVM-As would promote a progressive recovery of the host striatal DA terminal region function, which further supports their role in enhancing endogenous recovery mechanisms in the aged brain.

### tVM-As Rejuvenate the SN Microenvironment: Downmodulation of Microglial Pro-inflammatory Status

With age, both As and microglial cells become dysfunctional. As lose their neuroprotective, antioxidant, and pro-neurogenic potential, and microglia acquire a “primed” status (Streit, 2010; Njie et al., 2012; Niraula et al., 2017), capable of producing upregulated levels of pro-inflammatory cytokines when challenged with inflammatory/neurotoxic stimuli (L’Episcopo et al., 2010a,b, 2011c, 2018a,b). We thus questioned whether tVM-As might affect this “harmful” setting, thus ameliorating the injured microenvironment of middle-aged MPTP mice. To this end we first examined the effect of tVM-As on microglial response to MPTP. Notably, glial inflammatory mechanisms have long been recognized to contribute to both nigrostriatal degeneration and self-repair (see Marchetti and Abbracchio, 2005; Marchetti et al., 2005a,b,c; McGeer and McGeer, 2008; Marchetti et al., 2011; Przedborski, 2010; Gao et al., 2011; L’Episcopo et al., 2018a,b). We thus examined the microglial cell number and phenotype *in vivo* (Figures 5A–D) and found that tVM-As induced a significant (*p* ≤ 0.01) counteraction of MPTP-induced increased IBA1^+^ microglial cells (Figure 5B) displaying the morphology of activated macrophage-like microglia (Figures 5A1,A2). Hence, in MPTP/PBS middle-aged mice, we observed a greater number of stage 3 IBA1^+^, ameboid microglia, showing a round-shaped body with short, thick, and stout processes, or stage 4 phagocytic glial cells (Supplementary Table S3), with round-shaped cells and no processes, indicative of an M1-activated phenotype (Kreutzberg, 1996). In stark contrast, MPTP/tVM-As mice displayed a ramified, more quiescent phenotype, with elongated-shaped cell bodies and long and thicker processes, comparable to stages 1–2 (Figures 5A3,A4, Supplementary Table S3), which suggested a switch towards the M2 less-reactive phenotype. Accordingly, using qPCR, we found that the expression levels of the pro-inflammatory M1-cytokines *IL1-β*, *IL-6*, and *TNF-α* were significantly (*p* ≤ 0.01) upregulated by about 5- to 6-fold in MPTP/PBS, as compared to saline-injected controls, in the face of no changes of the low levels of expression of the anti-inflammatory M2-cytokine, *IL-10* (Figure 5C). By contrast, tVM-As significantly (*p* ≤ 0.01) reduced *IL1-β*, *IL-6*, and *TNF-α* to values almost comparable to saline/PBS mice, while *IL-10* gene expression increased significantly (*p* ≤ 0.05) at both 2 and 4 wpt (Figure 5D). Supporting these results, when the cytokine protein levels were determined in VM tissues of the different groups at 4 wpt, by ELISA, we found significantly (*p* ≤ 0.01) greater levels of *IL1-β*, *IL-6*, and *TNF-α* in MPTP/PBS as compared to MPTP/tVM-As treated mice, exhibiting cytokine values comparable to those determined in saline/PBS-injected mice (Figure 5E). Additionally, MPTP/tVM-As mice, but not MPTP/PBS mice, had increased IL-10 protein levels (Figure 5E).

Thus, tVM-As efficiently override the microglial pro-inflammatory status and reduce the number of activated IBA1^+^ cells within the middle-aged MPTP-lesioned SNpc during the studied experimental period. This effect was associated with a significant downregulation of the pro-inflammatory cytokines, TNF-α, IL-6, and IL-1β, associated to an increase of the anti-inflammatory cytokine, IL-10, at both gene and protein levels.

### tVM-As Upregulate As Antioxidant Self-defense *in vivo and ex vivo*: Contribution of Wnt/β-Catenin Signaling

Given the central role of As in antioxidant self-defense brain functions, we then looked at critical As-oxidative/nitrosative stress markers in SN tissues isolated from MPTP/PBS and MPTP/tVM-As, *in vivo* (Figure 6A). To differentiate the As-specific mRNAs vs. the SN-expressed mRNAs, we used *ex vivo* As cultures acutely isolated from MPTP/tVM-As and MPTP/PBS-As at 1 wpt (Figure 6B). Nrf2 is a conserved basic leucine zipper transcription factor which affords cytoprotection against xenobiotics and ROS through induction of antioxidant (ARE) and electrophile (EpRE) response elements (see Tebay et al., 2015; Zhang et al., 2017). HO1 is a principal mediator of cellular adaptive (i.e., antioxidant and anti-inflammatory) responses (Chen et al., 2009; Surh et al., 2009). SOD1 is critical for enhancing antioxidant self-defense during aging and neurodegenerative conditions, whereas its deficiency results in an accelerating aging phenotype (see Zhang et al., 2017).

Hence, we found that in response to MPTP, *Nrf2* and the antioxidant gene, *HO1*, but not *HO2*, were sharply upregulated (*p* ≤ 0.01), together with *SOD1*, in aging MPTP/tVM-As–grafted vs. MPTP/PBS mice, which instead failed to activate an antioxidant self-defense response to the MPTP challenge (Figures 6A,B). Notably, tVM-astros greatly (*p* ≤ 0.01) increased Nrf2-antioxidant genes as compared to saline/PBS mice exhibiting very low transcript levels (Figures 6A,B). Within the nicotinamide adenine dinucleotide phosphate (NADPH) oxidases, Nox2 is the predominant oxidase family member expressed in As, at both the mRNA and protein level (Belarbi et al., 2017). Here, we detected decreased activation/expression of *Nox2* in SN tissues from MPTP/tVM-As when compared to MPTP/PBS counterparts (Figures 6A,B) exhibiting an almost 3-fold increase over saline/PBS-injected mice. Additionally, tVM-As significantly counteracted the exacerbated expression of the harmful pro-inflammatory mediator, *iNOS*, vs. MPTP/PBS, showing instead a 3-fold upregulation (*p* ≤ 0.01, vs. saline-injected mice; Figures 6A,B). Remarkably, dual immunofluorescent stainings evidenced the neuroprotective effect of tVM-As and close interactions with both TH neurons and microglial cells, as suggested by the long GFAP^+^ processes close to the rescued TH^+^ neuronal cell body (Figures 6D1,D2), and in close contact with the long, ramified IBA1^+^ processes (Figures 6D4,D5). On the other hand, a severe neuronal loss, poor GFAP^+^–TH^+^ neuron interactions (Figures 6C1,C2) were observed in MPTP/PBS, where only round-shaped IBA1^+^ cells were seen close to the reactive GFAP^+^ As (Figure 6C6), thus supporting the failure of middle-aged MPTP-injured As to mount a neuroprotective response against MPTP.

Consistently, dual staining with GFAP and HO1 followed by confocal laser microscopic analyses uncovered an abundant co-expression of HO1 in tVM-As (*p* ≤ 0.01, vs. saline- and MPTP-injected mice) as determined by the sharp increase in the percentage of GFAP^+^/HO1^+^ out of the total GFAP^+^ cells (77 ± 11%) in MPTP/tVM-As, when compared to MPTP/PBS (20 ± 4%) and saline/PBS mice (12 ± 4%; Figure 6C3). These results coupled to the abundant expression of iNOS in GFAP^+^ As of MPTP/PBS (Figure 6C5) as opposed to MPTP/tVM-As mice suggested failure of aged As to activate the antioxidant and anti-inflammatory response upon MPTP challenge. Given that when iNOS and NADPH oxidase are present together, a potent toxin, peroxynitrite (ONOO-), may be generated which promotes the nitration of proteins (Gao et al., 2011), we then looked at the colocalization of 3-NT and found an abundant expression of 3-NT in GFAP^+^ As of MPTP/PBS (Figure 6C4) but not of MPTP/tVM-As (not shown). These findings suggested the potential of tVM-As to ameliorate the exacerbated endogenous oxidative/nitrosative glial status of the aged injured SN milieu *via* an upregulation of antioxidant functions.

As a proof of concept, we next studied some functional properties of tVM-As derived from MPTP/PBS and MPTP/tVM-As mice, looking at the mitochondrial activity with the MTT assay, the production of ROS with the redox membrane-permeant probe DCFH-DA, and the generation of iNOS-derived NO, in cell free supernatant using the Griess reagent (Figures 6E–G). To study the effect of inhibition of oxidative and nitrosative stress mediators, the freshly prepared GFAP^+^ cells were cultured in the absence or the presence the specific iNOS inhibitor, L-Nil (Marchetti et al., 2002; Morale et al., 2004), whereas to study the effect of an exogenous inflammatory trigger, lipopolysaccharide (LPS) was applied at a dose of 100 ng/ml. Finally, the effect of Wnt/β-catenin activation was studied using AR, a drug that antagonizes GSK-3β (i.e., the kinase that phosphorylates β-catenin, leading to its degradation).

First we found that mitochondrial activity was increased in tVM-As when compared to MPTP/PBS-As metabolic activity, showing significantly (*p* ≤ 0.01) reduced activity vs. saline/PBS-As (Figure 6G). This effect is in line with the reduced amounts of both ROS and reactive nitrogen species (RNS) produced by MPTP/tVM-As vs. MPTP/PBS (Figures 6E,F), exhibiting significantly (*p* ≤ 0.01) greater levels vs. saline/PBS-As. These results thus support increased antioxidant properties of tVM-As. Accordingly, the specific iNOS-NO inhibitor, L-Nil, efficiently counteracted the sharp increase in oxidative and nitrosative stress of MPTP/PBS-As vs. MPTP/tVM-As counterparts (Figures 6F,G), with a beneficial effect on mitochondrial reductase activity (Figure 6E). Conversely, application of LPS counteracted the low ROS and RNS levels of MPTP/tVM-As, reducing mitochondrial activity, and further increased the already elevated ROS and RNS of MPTP/PBS-As (Figures 6E–G).

Given the decline of As-derived Wnts with age, we asked whether Wnt/β-catenin signaling activation might affect the exacerbated redox status of MPTP/PBS-As. Remarkably, we found a significant counteraction of both ROS and RNS upregulation of MPTP/PBS-As in AR-treated vs. untreated As cultures, to levels almost comparable to those measured in tVM-As (Figures 6F,G), which resulted in increased metabolic activity (Figure 6E).

Together, tVM-As grafts downregulate the exacerbated oxidative/nitrosative status of the aged MPTP-injured SNpc and As cultures *via* an increase of Nrf2-antioxidant genes, thereby shifting the pro-inflammatory and oxidative SN microenvironment. Additionally, the ability of Wnt/β-catenin activation to revert ROS- and RNS-exacerbated production of MPTP/PBS-As further suggested the contribution of Wnt/β-catenin signaling in redox properties of VM-As.

### tVM-As Promote Upregulation of Wnt/β-Catenin Genes and Protect DAergic Neurons Against MPTP/MPP^+^: Crosstalk With Oxidative Stress Pathways

**Figure 7:**
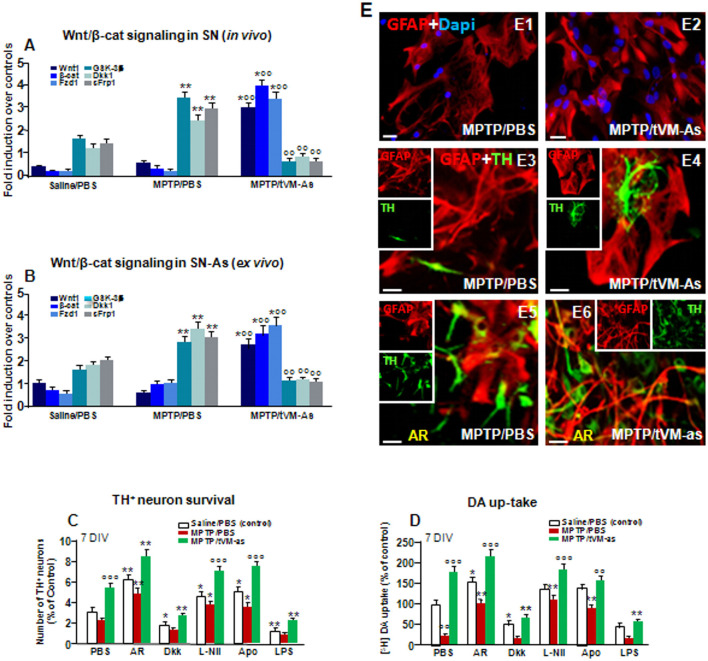
tVM-As grafts promote activation of Wnt/β-catenin signaling in SN tissue and As and reverse the impaired DAn growth *in vitro*. **(A,B)** Wnt/β-catenin signaling transcripts were analyzed by quantitative real-time PCR (qPCR) in SNpc tissues derived from MPTP/PBS- and MPTP/tVM-As mice, *in vivo* **(A)**, and acutely isolated As, *ex vivo*, from both groups **(B)**. Values (AU, mean % ± SEM of *n* = 5 samples/experimental group) are expressed as fold changes over control. VM-As grafts upregulated *Wnt1*, *β-catenin*, and *Fzd1 receptor*, whereas the endogenous Wnt antagonist*s, Dkk1, sFrp, and GSK-3β* are downregulated vs. MPTP/PBS in both SN tissues **(A)** and As-derived cultures **(B)** at 1 wpi. **p ≤ 0.01 vs. saline/PBS; °°*p* ≤ 0.01 vs. MPTP/PBS; ^*°^°*p* ≤ 0.01 vs. saline/PBS and MPTP/PBS, within each treatment group, respectively, by ANOVA followed by *post hoc* Newman–Keuls test. **(C,D)** Quantification of TH^+^ neuron number at 7 DIV **(C)** and DA uptake levels measured by [^3^H]DA incorporation **(D)** in a direct coculture paradigm between MPTP/PBS- and MPTP/tVM-As acutely isolated, *ex vivo*, and primary mesencephalic DAn grown on the top of As, in the absence or the presence of the GSK-3β inhibitor AR, the Wnt antagonist Dkk1, the antioxidant apocynin (Apo), or the specific iNOS inhibitor L-Nil. Wnt signaling activation with AR increased TH neuron number and DA uptake levels in MPTP/PBS-As–DAn coculture **(C–D,E5)** and significantly magnified TH neuron number and functionality of MPTP/tVM-As–DAn coculture **(C–D,E6)**. Lowering oxidative and nitrosative stress with Apo and L-Nil also increased TH neuron number and DA uptake in MPTP/PBS-As–DAn coculture, albeit to a lower extent vs. their tVM-As–DAn treated counterparts **(C,D)**. Mean ± SEM values (*n* = 3 different culture preparations) are reported. *p ≤ 0.05, **p ≤ 0.01 vs. saline/PBS control; °°*p* ≤ 0.01 vs. PBS; °°°*p* ≤ 0.01 vs. all treatments, within each group, respectively, by ANOVA followed by *post hoc* Newman–Keuls test. **(E1–E6)** Confocal images of GFAP^+^ (*red*) in As cultures from MPTP/PBS **(E1)** and MPTP/tVM-As **(E2)**, and TH neurons (green) and GFAP (red) in the coculture from MPTP/PBS- **(E3)** and MPTP/tVM-As **(E4)**, showing TH+ neuron growth at 5–7 DIV neurons in the presence of tVM-As **(E4)**, compared to the impaired TH neuron development in MPTP/PBS-As coculture **(E3)**. Activation of Wnt/β-catenin signaling improved TH neuron growth in MPTP/PBS-As **(E5)**, an effect that was magnified in MPTP/tVM-As–DAn coculture **(E6)**. *Bars*: **(E1,E2)**, 23 μm; **(E3–E6)**, 20 μm.

Previous findings clearly indicated that the aging process is associated with a sharp decline of As Wnts in the face of an increase of endogenous Wnt antagonists (L’Episcopo et al., 2011a,b, 2013, 2014b; Okamoto et al., 2011; Seib et al., 2013; Orellana et al., 2015; reviewed in Marchetti, 2018). We thus hypothesized that an As-driven disbalance of the *Nrf2-ARE* axis in middle-aged mice might contribute to aging-induced loss of Wnt signaling, and looked at the ability of tVM-As to override this hostile Wnt setting. Hence, both SNpc tissues from tVM-As (Figure 7A) and tVM-As–derived cultures (Figure 7B) enriched the expression of Wnt signature genes (*p* ≤ 0.01), including *Wnt1* and *β-catenin*, together with *Fzd-1* receptor, vs. MPTP/PBS SNpc tissues and MPTP/PBS-As cultures (Figures 7A,B). By contrast, a number of endogenous Wnt signaling inhibitors, including the *Dkk1, sFrp1*, and *GSK-3β*, were markedly (*p* ≤ 0.01) downregulated by 1 wpt in tVM-As, compared to MPTP/PBS exhibiting significantly (*p* ≤ 0.01) increased levels compared to saline-injected controls (Figures 7A,B), suggesting that tVM-As promoted a switch of the redox- and inflammation-sensitive Wnt/β-catenin signaling response. Hence, by reverting the age-dependent decline of endogenous Wnt tone, this, in turn, might enhance the ensuing DAergic neuronal survival. To test this hypothesis, primary (E18) mesencephalic TH^+^ neurons were grown on the top of MPTP/PBS-As (Figure 7E1) or tVM-As (Figure 7E2), for 5–7 DIV, and the survival and growth of TH^+^ neurons as well as their functionality were studied using immunocytochemistry and DA uptake assay (Figures 7C,D). Hence, we found increased TH^+^ neuron numbers and increased neurite extension and DA uptake levels when cocultured with tVM-As (Figures 7C,D,E3,E4). By contrast, TH^+^ neurons grown on the top of MPTP/PBS-As exhibited a marked inhibition of neuronal survival, growth and functionality (Figures 7C,D,E3,E4). Accordingly, activating Wnt/β-catenin signaling with AR by lowering ROS and RNS and ameliorating MPTP/PBS-As metabolic activity (see Figures 6E–G) in turn increased both TH^+^ neuron survival and DA uptake in MPTP/PBS-As cocultures (Figures 7C,D,E5), and further increased both parameters in the tVM-As–neuronal cocultures (Figures 7C,D,E6). By contrast, antagonizing Wnt/β-catenin signaling by the exogenous application of Dkk1 to tVM-As coculture reduced both neuronal numbers and function, thereby supporting increased As-Wnt tone as a crucial event involved in DAn growth. On the other hand, in MPTP/PBS-As overexpressing Dkk1, exogenous Dkk1 only slightly affected the already inhibited neuronal numbers found in MPTP/PBS-As–neuron cultures (Figures 7C,D). Finally, lowering ROS and RNS by the application of the antioxidant Apo, or L-Nil, in MPTP/PBS-As increased the number of TH^+^ neurons and DA uptake levels vs. the untreated cocultures (*p* ≤ 0.05), but these effects were significantly (*p* ≤0.05) lower when compared to their tVM/As counterparts, where L-Nil and Apo induced a further increase in neuronal growth and DA uptake vs. the untreated tVM-As–neuronal cocultures (Figures 7C,D).

Together, these data suggest the potential of grafted VM-As to reverse the As decline of canonical Wnt’s signaling components, downregulate major Wnt antagonists, and trigger the activation of an *Nrf2/Hmox/Wnt/β-catenin* axis favoring the “rejuvenation” of the aged, injured microenvironment, likely contributing to the DAergic neuroprotection and neurorescue herein observed.

## Discussion

Given that aging is the leading risk factor for PD development, and that by middle age, several parameters of astroglial and DAergic neuronal functionality start to be impaired, we focused on the middle-aged (9–11 months) male midbrain microenvironment to address the ability of primary fully differentiated tVM-As to activate intrinsic mechanisms that might rejuvenate As–neuronal dialog and promote DAergic neurorepair in MPTP-induced DAergic neurotoxicity. We herein report for the first time that grafting VM-As derived from postnatal (P2–3) midbrain within the middle-aged MPTP-injured SN can trigger a significant time-dependent endogenous nigrostriatal DAergic neurorescue. Clearly, various interactions between exogenous tVM-As and the pathological host milieu may underlie the improvement herein observed, and further time-course studies and in-depth molecular analyses both at a tissue and at a single-cell level are clearly needed to unravel how tVM-As grafts may drive a DAergic neurorescue program. However, from the presented results and based on our previous (see L’Episcopo et al., 2012, 2013, 2014b, 2018a, b; and as reviewed by Marchetti, 2018) and the recent literature findings (Tebay et al., 2015; Zhang et al., 2015, 2017; Zheng et al., 2017; Rizor et al., 2019), it seems tempting to speculate that the observed changes might result from a beneficial tVM-As–to–host SN crosstalk, promoting the rejuvenation of the host microenvironment, through the activation of an astrocytic *Nrf2/ARE/Wnt/β-catenin* prosurvival axis.

Notably, complex mechanisms are responsible for SNpc-DAergic cell death in PD, where the demise of this mesencephalic neuronal population is a process that is very long and is still not yet clarified (Del Tredici and Braak, 2012), as opposed to MPTP-induced DAergic degeneration (Langston, 2017), and extrapolations must be very careful. In the present study, our approach was aimed at combining: (i) the effect of age with; (ii) neurotoxin exposure; (iii) the consequent exacerbated inflammation; and (iv) male gender (representing four strong risk factors for PD), to address the ability of As grafting to mitigate the SN microenvironment and DAergic cell death.

Here, we found that, within the aged microenvironment, the proliferating tVM-As expressed S100β, but not microglia or neuronal markers, and survived for at least 4 wpt. At the SN level, tVM-As counteracted aging and MPTP-induced DAergic cell body loss, and at the striatal level, tVM-As promoted the recovery of DAT- and TH-IR, which was corroborated by the increased striatal synaptosomal DA uptake capacity and the reversal of motor impairment of aged MPTP mice, indicating the ability of tVM-As to restore nigrostriatal DAergic neurons and reinstate DAergic functionality at least for the studied time window of 4 wpt; further studies are needed to confirm, at a protein level, the capacity of tVM-As to revert the downregulation of the studied DA-signaling genes, as observed by qPCR analyses.

By analyzing microglial cells, we found that tVM-As efficiently counteracted aging and MPTP-induced increase of microglial cell number within the injured midbrain and shifted microglial morphological appearance from the M1 macrophage–like to the M2 more quiescent glial phenotype. Especially, in MPTP/PBS mice, glial cells showed round-shaped bodies with either short and thick processes or no processes, as observed in activated microglial cells (Kreutzberg, 1996), whereas in tVM-As mice, microglial cells exhibited more elongated cell bodies and longer, ramified processes, similar to the M2 glial phenotype. Accordingly, tVM-astros reversed the upregulated levels of the pro-inflammatory M1 cytokines, TNF-α, IL-6, and IL1-β, at both gene and protein expression levels in SN tissues, as compared to MPTP/PBS mice, but significantly increased the anti-inflammatory cytokine, IL-10, therefore supporting tVM-As ability to downregulate microglial exacerbation of aged MPTP/PBS mice. While further studies are clearly needed to address tVM-As–microglial interactions, their close contacts coupled to the observed change in glial phenotype may favor the possibility of a “beneficial” As–microglia crosstalk in tVM-As–grafted vs. MPTP/PBS mice, likely contributing to the downregulation of inflammation and DAergic neurorescue. In stark contrast, when the microglia is chronically activated, as observed in MPTP/PBS aging mice, As can lose both immunomodulatory and neuroprotective properties with harmful consequences for the dysfunctional DAergic neurons, as herein observed.

We then focused on the astrocytic compartment and found that tVM-As grafts sharply increased the expression of *Nrf2*, a chief astrocytic regulator of oxidative stress and inflammation, and upregulated the antioxidant and anti-inflammatory HO1 and *SOD1* transcripts, in the face of a marked suppression of *iNOS*. Clearly, from our gene expression analysis, it is not possible to differentiate the exogenous tVM-As vs. the endogenous As-expressed mRNAs, albeit using *ex vivo* As cultures from MPTP/tVM-As and MPTP/PBS-As at 1 wpt, we delineated a clear upregulation of antioxidant and Wnt signaling genes in MPTP/tVM-As vs. their MPTP/PBS-As counterparts. Especially, we found VM-As of middle-aged MPTP-lesioned mice as a critical source of Wnt antagonists likely responsible for their failure to exert neuroprotection. Hence, at a functional level, tVM-As generated significantly lower amounts of ROS and RNS compared to the exacerbated reactive mediators of MPTP/PBS-As, resulting in As mitochondrial impairment, whereas Wnt signaling activation in MPTP/PBS-As efficiently reversed the upregulated ROS and RNS to levels measured in L-Nil–treated As levels, suggesting crosstalk between Wnt signaling and oxidative stress pathways. Remarkably, tVM-astros promoted Wnt/β-catenin activation within the middle-aged, MPTP-injured SN *in vivo* and in *ex vivo* direct As–neuron coculture paradigms, where MPTP/tVM-As promoted the growth and functionality of developing primary mesencephalic DAn, as compared to their counterparts derived from MPTP/PBS mice, which failed to exhibit the critical supportive properties and instead inhibited TH^+^ neuron survival and functionality.

Thus, switching the SN neurorescue-unfriendly environment, grafted As promoted a beneficial antioxidant/anti-inflammatory “*Wnt-on*” prosurvival milieu, highlighting *As*-derived factors/mechanisms as the crucial keys for successful therapeutic outcomes in PD.

### As Grafting Switched the Harmful SN Milieu of Aging MPTP Mice Driving the Master Regulator of the Oxidative Stress and Inflammatory Response

With advancing age, the decline of the nigrostriatal DAergic system coupled with the progressive loss of DAergic neuron adaptive potential is believed to contribute to the slow nigrostriatal degeneration of PD (Hornykiewicz, 1993; Bezard et al., 2000; Collier et al., 2007; de la Fuente-Fernández et al., 2011; Blesa et al., 2017). In fact, the activation of endogenous compensatory mechanisms is thought to mask the appearance of PD before the appearance of the first clinical symptoms (Zigmond et al., 2009; Blesa et al., 2017), which raises the possibility that some individuals with PD suffer from a reduction of these neuroprotective mechanisms and that treatments that boost these mechanisms may provide therapeutic benefit (see Zigmond et al., 2009). Interestingly, while young adult rodents experience a time-dependent recovery/repair from neurotoxic or immunological challenges, aging mice fail to recover for their entire life span (L’Episcopo et al., 2011a,b,c). With aging, the glial adaptive mechanisms are reduced, resulting in increased DAergic neuron vulnerability to various risk factors, including genetic, inflammatory, and environmental toxic exposures, such as MPTP (L’Episcopo et al., 2018a).

In fact, declined Nrf2-antioxidant signaling during aging leads to accumulation of ROS/RNS and oxidative stress, which is either causally linked or associated with numerous health problems including neurodegenerative conditions; thus, targeting Nrf2 has been suggested as a promising therapeutical avenue in neurodegeneration (Abdalkader et al., 2018). Remarkably, previous studies of Chen et al. (2009) indicated that *Nrf2* expression restricted to As is sufficient to protect against MPTP toxicity, suggesting that As modulation of the Nrf2-ARE pathway is a promising target for therapeutics aimed at reducing or preventing neuronal death in PD (Chen et al., 2009). Significantly, Lastres-Becker et al. (2012) reported that α-synuclein expression and Nrf2 deficiency cooperate to aggravate protein aggregation, neuronal death, and inflammation in early-stage PD, and very recent findings further define As oxidative/nitrosative stress as a key etiopathogenetic factor in PD (Rizor et al., 2019). Additionally, we pinpointed that by middle age, a significant decrease of *Nrf2/HO1* response to MPTP in striatal and SVZ-As played a prominent role and synergized with the heightened inflammatory SVZ milieu to downregulate SVZ neurogenesis (L’Episcopo et al., 2013). Here, we further uncovered that VM-As grafts promoted a switch of inflammatory M1 microglia phenotype and turned the aged As into a supportive and neuroprotective A2 phenotype.

In fact, amongst glial cytotoxic molecules, iNOS-derived NO, a superoxide from the plasma membrane NADPH oxidase, associated with a number of potent inflammatory cytokines, including TNF-α, IL-1β, IL-6, and IFN-γ, is known to exert detrimental effects in DAergic neurons (see Gao and Hong, 2008; Hirsch and Hunot, 2009). Indeed, when iNOS and NADPH oxidase are present together, a potent toxin, ONOO-, may be generated, which promotes the nitration of proteins (Gao and Hong, 2008), with harmful consequences for DAergic neurons. Conversely, As-secreted antioxidant factors represent a critical self-protective system against MPTP and 6-OHDA cytotoxicity (Chen et al., 2009). In fact, the prolonged dysfunction of As and activation of microglia accelerate degeneration of DAergic neurons in the rat SN and block compensation of early motor dysfunction induced by 6-OHDA (Kuter et al., 2018).

Here, we uncovered the ability of VM-As grafts to override the strong oxidant and pro-inflammatory status of the SN milieu of middle-aged MPTP-injured As overexpressing iNOS and the harmful RNS footprint, 3NT, by decreasing the number activated macrophage-like microglia and the expression of major inflammatory transcripts and proteins, *in vivo*, resulting in suppression of the exacerbated ROS and RNS production of MPTP-injured As, *ex vivo*.

Coupled to the critical role of As mitigating mitochondrial dysfunctions in human DAergic neurons derived from iPSC (Du et al., 2018), these present findings support the ability of VM-As grafts to boost antioxidant self-defenses, as a potential mechanistic link in the herein observed DAergic neurorescue effects.

### As Grafting Triggering Wnt/Nrf2/HO1 Crosstalk Mitigates Inflammation Promoting DAn Neurorescue

The Wnt/β-catenin signaling pathway is of utmost importance owing to its ability to promote tissue repair and regeneration of stem cell activity in diverse organs, and in light of its crucial role in age-related pathogenesis and therapy of disease (Nusse and Clevers, 2017; García-Velázquez and Arias, 2017; Marchetti et al., 2020). In the last decade, we characterized As-derived *Wnt1* as crucial actor in As–DAn crosstalk involved in neuroprotection against several neurotoxic and inflammatory insults (Marchetti and Pluchino, 2013; Marchetti et al., 2013; Marchetti, 2018) and the regulation of neurogenesis and SVZ plasticity *via* crosstalk with inflammatory and oxidative stress signaling pathways during aging and MPTP-induced parkinsonism (Marchetti et al., 2020).

Indeed, a major finding of aging is the decline of Wnt signaling and that this “*Wnt-off*” state very likely drives the decline of neurogenesis and the exacerbation of inflammation, thus increasing DAn vulnerability to a number of cytotoxic and inflammatory insults (Marchetti and Pluchino, 2013; L’Episcopo et al., 2018a,b; Marchetti, 2018). Here, we further unveiled that VM-As grafts promoted an enriched expression of canonical *Wnt* signature genes in the middle-aged MPTP-injured VM, which included *Wnt1*, *β-catenin*, and *Fzd1* receptor, thus triggering a *Wnt-on* state, likely contributing to DAergic neurorescue. Accordingly, a number of endogenous Wnt signaling antagonists, such as *GSK-3β, Dkk1, and sFrp1*, were downregulated by 1 wpt in tVM-As SN tissues and As cultures, when compared to their MPTP/PBS-As counterparts exhibiting a significant upregulation of endogenous Wnt antagonists, supporting the *Wnt-off* state of the aged MPTP-injured midbrain, as observed in our previous studies (Marchetti, 2018). Here, As overexpression of Wnt antagonists and upregulated ROS and RNS levels of MPTP/PBS mice impaired As–neuron interactions *in vivo* and *ex vivo*, thus resulting in a marked inhibition of DAn survival and growth, whereas activating Wnt/β-catenin signaling, as observed in VM-As–grafted mice and VM-As–derived cultures, or MPTP/PBS-As–derived cultures treated with the GSK-3β antagonist, powerfully reverted oxidative/nitrosative stress markers promoting DAn survival and growth.

Thus, in light of the role of Wnt signaling in the inflammatory and oxidative stress response and the crosstalk between inflammatory and Wnt signaling components (Chong et al., 2007, 2010; Halleskog et al., 2011; Kilander et al., 2011; L’Episcopo et al., 2011b; Schaale et al., 2011; Halleskog and Schulte, 2013; Marchetti and Pluchino, 2013; Ma and Hottiger, 2016; Zheng et al., 2017; L’Episcopo et al., 2018a,b; Marchetti, 2018), it seems tempting to suggest that the *Wnt-off* state of MPTP/PBS mice likely exacerbates the pro-inflammatory microglial phenotype of MPTP/PBS mice, in turn responsible for the hostile SN milieu that synergizes with the downregulation of MPTP/PBS-As antioxidant self-defenses.

In stark contrast, Wnt/β-catenin activation of tVM-As–grafted mice might well promote a beneficial effect, switching the microglial M1 phenotype to a likely more quiescent anti-inflammatory state (see L’Episcopo et al., 2018a,b). Especially, a lack of As-derived Wnt-microglial dialog might well contribute to the loss of major Nrf2-antioxidant genes responsible for As failure to protect and rescue/repair the injured DAn of middle-aged MPTP mice.

Together, these findings argue in favor of reciprocal As/microglial/neuron interactions and suggest the *Nrf2/HO1/Wnt/β-catenin* axis as a critical mediator in promoting neuroprotection.

## Conclusions

We have shown that grafting tVM-astros can override the aged hostile SN milieu and drive DAergic neurorescue in MPTP-induced nigrostriatal toxicity triggering the activation of a “beneficial” astrocytic *Nrf2/Hmox1/Wnt/β-catenin* axis that rejuvenates the SN microenvironment and favors DAergic neurorescue/neurorepair.

In light of the emerging implications of dysfunctional As in major human NDs (Endo et al., 2015; Anderson et al., 2016; L’Episcopo et al., 2016, 2018a,b; Booth et al., 2017; Patel et al., 2019), together with the critical role of As and As-derived factors in both pharmacological and cell therapeutical interventions (Kondo et al., 2014; Yang et al., 2014; Nicaise et al., 2015; Hall et al., 2017; Rivetti di Val Cervo et al., 2017; Barker et al., 2018; Du et al., 2018; Kuter et al., 2018; Song et al., 2018; Bali et al., 2019; Klapper et al., 2019; Rizor et al., 2019), our findings highlight As-focused therapies as the crucial key for beneficial outcomes in PD.

## Data Availability Statement

All datasets generated for this study are included in the article/Supplementary Material.

## Ethics Statement

This study has been approved by the local Ethics Committee “Comitato Etico IRCCS Sicilia-Oasi Maria SS” of the Associazione Oasi Maria SS., based in Troina (Italy), Via Conte Ruggero, 73.

## Author Contributions

Authors contributing to the presented experimental findings and manuscript editing. BM: conception and design, data analysis and interpretation, manuscript writing. MS: astrocyte transplantation, histopathology, data analyses and interpretation, manuscript writing and final approval of manuscript. CT, NT, FL’E, SC, and CG: performed experiments, data analyses and interpretation, final approval of manuscript.

## Supplementary Material

The Supplementary Material for this article can be found online at: https://www.frontiersin.org/articles/10.3389/fnagi.2020.00024/full#supplementary-material.









## Conflict of Interest

The authors declare that the research was conducted in the absence of any commercial or financial relationships that could be construed as a potential conflict of interest.
