# Corrigendum: Boosting antioxidant self-defenses by grafting astrocytes rejuvenates the aged microenvironment and mitigates nigrostriatal toxicity in parkinsonian brain via an Nrf2-driven Wnt/β-catenin prosurvival axis

**DOI:** 10.3389/fnagi.2025.1596878

**Published:** 2025-04-09

**Authors:** Maria Francesca Serapide, Francesca L'Episcopo, Cataldo Tirolo, Nunzio Testa, Salvatore Caniglia, Carmela Giachino, Bianca Marchetti

**Affiliations:** ^1^Pharmacology Section, Department of Biomedical and Biotechnological Sciences, Medical School, University of Catania, Catania, Italy; ^2^Section of Neuropharmacology, OASI Research Institute-IRCCS, Troina, Italy

**Keywords:** Parkinson's disease, aging, astrocyte–neuron crosstalk, neuroinflammation, dopaminergic neurons, neuroprotection

In the published article, there was an error in Figure 4 as published. The image in Figure 4 A2 was erroneously selected and should have instead illustrated the time-course effect of MPTP ± tVM-AS paralleling striatal DAT-IF and TH-IF image analyses (B, C), DA uptake levels (D), and behavioral analyses (E). The corrected Figure 4 and its caption appear below.

**Figure 4:**
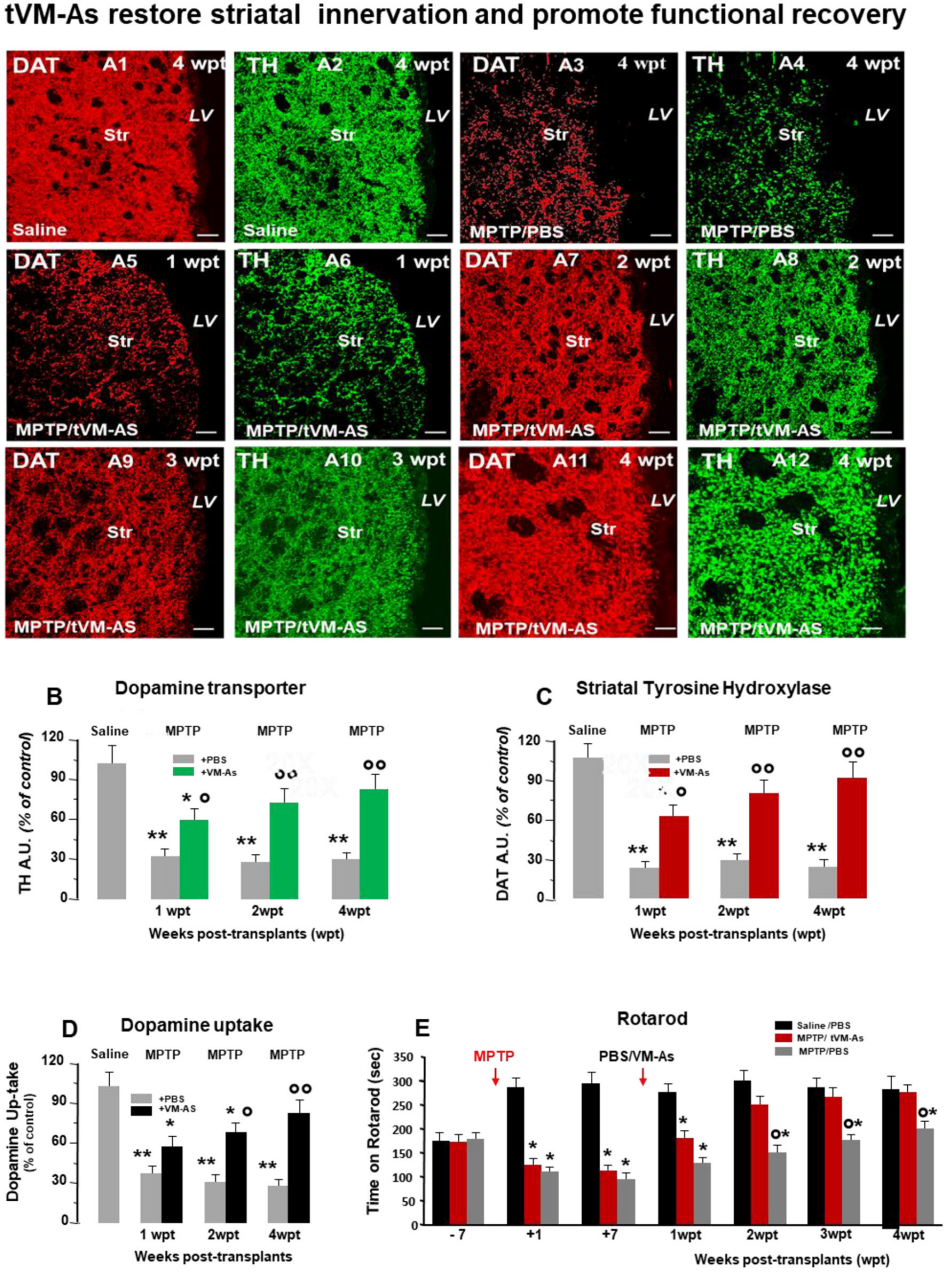
tVM-As grafts counteract MPTP-induced loss of DAergic innervation and synaptosomal dopamine (DA) uptake in the striatum, and revert Parkinson's disease (PD) motor deficits. **(A1–A12)** Representative confocal images of dopamine transporter (DAT, revealed in CY3, red)-fluorescence intensity (FI) and tyrosine hydroxylase (TH, revealed in FITC, green) showing the recognized loss of striatal DAT-IF and TH-IF 4 weeks post MPTP treatment compared to saline **(A1–A4)** as opposed to the ability of tVM-As to counteract MPTP-induced loss of DAT-IF and TH-IF at all time tested **(A5–A12)**. DAT **(B)** and TH-**(C)** immunofluorescent staining measured by image analysis. Scale bars: 50 μm. **(D)** VM-As grafts increase high-affinity striatal (Str) DA uptake assessed by [3H]DA incorporation (mean % SEM). **(E)** Motor performances on rotarod showing recovery from motor impairment in MPTP/tVM-As but not MPTP/PBS mice. ^*^p < 0.05, ^**^p < 0.01 vs. saline/PBS; ^°^*p* < 0.05, ^°°^*p* < 0.01 vs. MPTP/PBS, at each time interval respectively, by ANOVA followed by *post hoc* Newman–Keuls test.

In the published article, there was an error in Figure 5 as published. The image in Figure 5 A2 was also erroneously selected. The corrected Figure 5 and its caption appear below.

**Figure 5:**
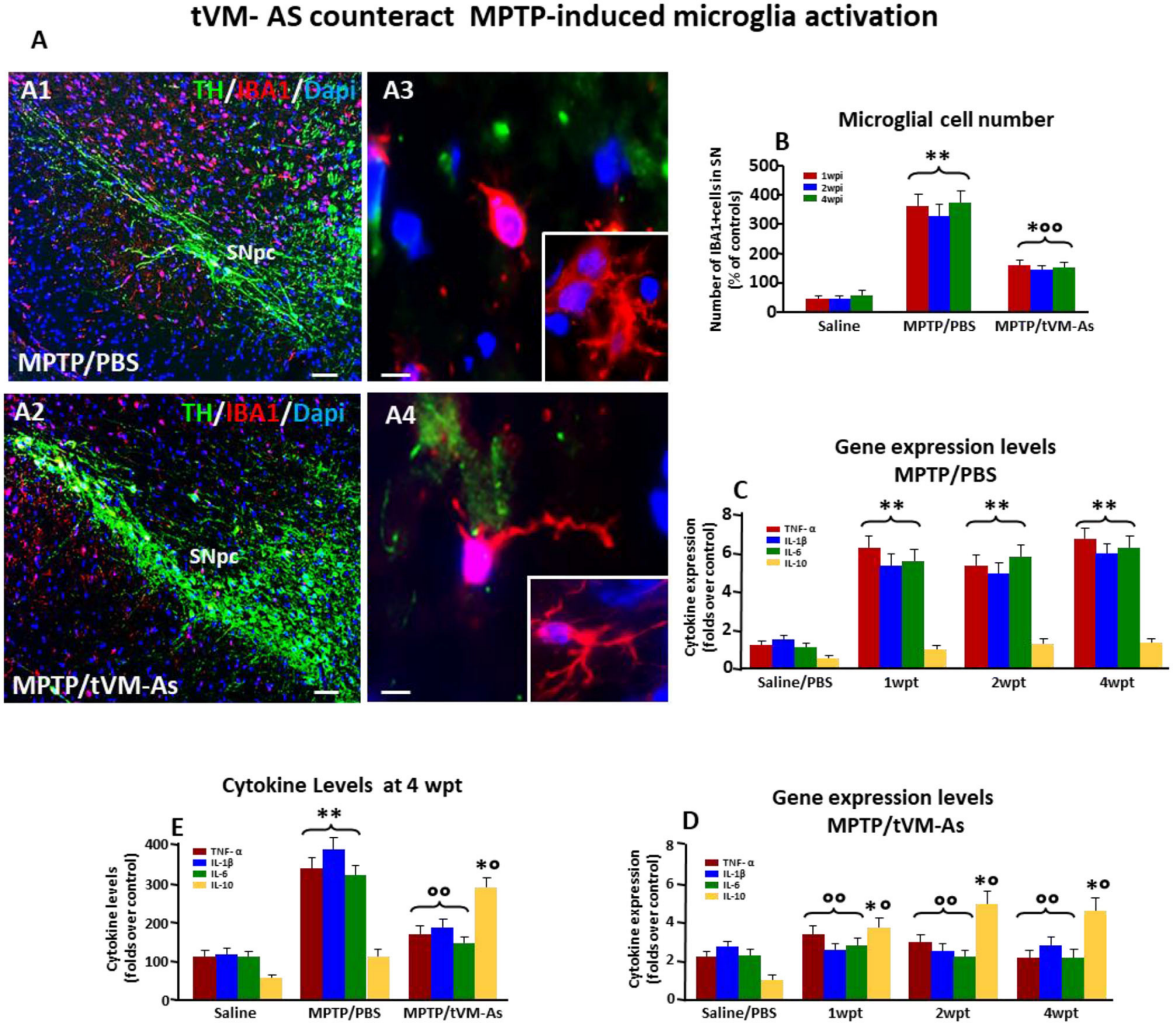
tVM-As downregulate microglial pro-inflammatory phenotype in SNpc. **(A, B)** tVM-As reverse MPTP-induced reactive microglial cells displaying the morphology of activated macrophage-like microglia **(A1,A3)** and the increased IBA1+/Dapi+ microglial cell numbers in midbrain sections at the level of the SNpc **(B)**. Note the ramified microglia in SNpc of tVM-As mice **(A2, A4)**. Scale bars: **(A1, A2)**, 100 μm; **(A3, A4)**, 25 μm. **(C, D)** SNpc tissues were processed for gene expression analyses of mRNA species using qRT-PCR. Values (AU, mean % SEM of *n* = 5 samples/experimental group) are expressed as fold changes. In MPTP/PBS, inflammatory (TNF-α, IL1-β, IL-6) mRNAs are upregulated by about 5- to 6-fold (*p* < 0.01) over saline-injected controls **(C)**, whereas the anti-inflammatory cytokine IL-10 is not affected. Transplantation of VM-As in MPTP mice induced a significant (*p* < 0.01) downregulation of pro-inflammatory markers at all tps but increased IL-10 expression *vs*. MPTP/PBS **(D)**. **(E)** Evaluation of IL-1β, TNF-α, IL-6, and IL-10 at a protein level, as determined by enzyme-linked immunosorbent assay (ELISA) in homogenate tissue samples (mean % SEM of *n* = 5 samples/experimental group), documents the ability of tVM-As to suppress the pro-inflammatory cytokines in the face of a significant increase in the anti-inflammatory cytokine, IL-10, when levels are compared to MPTP/PBS mice. ^*^p < 0.05, ^**^p < 0.01 vs. saline/PBS; ^°°^*p* < 0.01 vs. MPTP/PBS; ^*°^*p* < 0.01 vs. saline/PBS and MPTP/PBS, at each time interval respectively, by ANOVA with post hoc Newman–Keuls.

In the published article, in the section **Results**, subsection *tVM-As Grafts Counteract MPTP-Induced Loss of DAergic Innervation and Synaptosomal DA Uptake in the Str and Revert PD Motor Deficits*, paragraph two, a text correction is needed to comment on the corrected Figure 4.

This sentence previously stated:

“Hence, tVM-As grafts efficiently counteracted the MPTP-induced loss of striatal TH and DAT innervation (Figures 4A1–A3 and Figures 4B–C).”

The corrected sentence appears below:

“Hence, tVM-As grafts efficiently counteracted the MPTP-induced loss of striatal TH and DAT innervation (Figures 4A1–A12 and Figures 4B–C).”

The authors apologize for these errors and state that they do not change the scientific conclusions of the article in any way. The original article has been updated.

