# Supplementary material for: Boosting Antioxidant Self-defenses by Grafting Astrocytes Rejuvenates the Aged Microenvironment and Mitigates Nigrostriatal Toxicity in Parkinsonian Brain via an Nrf2-Driven Wnt/β-Catenin Prosurvival Axis
Source: Front Aging Neurosci. 2020 Mar 12;12:24. doi: 10.3389/fnagi.2020.00024 (PMC7081734; doi:10.3389/fnagi.2020.00024)
Supplement: Supplementary file 1 [file Table_1.DOCX]

**Supplementary Table 1.** List of antibodies used for immunohistochemistry and cytochemistry

| **Abs** | **SOURCE** | **Diluition** |
| --- | --- | --- |
| Ms anti-Nestin | Chemicon | 1:500 |
| Ms anti-Olig2 | Millipore | 1:100 |
| Rb anti-GFAP | Chemicon | 1:1000 |
| Rb anti-GFAP | DAKO | 1:250 |
| Ms anti-GFAP | Sigma | 1:250 |
| Rt anti-BrdU | Abcam | 1:300 |
| Ms anti-BrdU | Sigma | 1:100 |
| Ms anti-BrdU | DAKO | 1:200 |
| Rt anti-PCNA | Santa Cruz B. | 1:200 |
| Rb anti-TH | Pel-Freez | 1:200 |
| Rb anti-TH | Chemicon | 1:200 |
| Ms anti TH | Boehringer Mannheim | 1:200 |
| Shp anti TH | Pel-Freez | 1:200 |
| Rt anti-Dat | Chemicon | 1:200 |
|  |  |  |
