# Supplementary material for: Boosting Antioxidant Self-defenses by Grafting Astrocytes Rejuvenates the Aged Microenvironment and Mitigates Nigrostriatal Toxicity in Parkinsonian Brain via an Nrf2-Driven Wnt/β-Catenin Prosurvival Axis
Source: Front Aging Neurosci. 2020 Mar 12;12:24. doi: 10.3389/fnagi.2020.00024 (PMC7081734; doi:10.3389/fnagi.2020.00024)
Supplement: Supplementary file 2 [file Table_2.DOCX]

**Supplementary Table 2.** List of probes and IDs used for quantitative Real time PCR

| **PROBES** | **IDs** |
| --- | --- |
| Ctnnb1/ Beta-catenin | Mm00483039-m1 |
| Dkk1 | Mm00438422-m1 |
| Fzd1 | Mm 00445405-s1 |
| Gfap | Mm 00546086-m1 |
| Gp91phox/Nox2 | Mm01287743-m1 |
| Gsk3b | Mm00444911-m1 |
| Hmox1 | Mm00516005-m1 |
| Hmox2 | Mm00468922-m1 |
| iNos2 | Mm00440485-m1 |
| Nrf2 | Mm00477784-m1 |
| Slc6a3 | Mm 00438388-m1 |
| Sfrp2 | Mm01213947-m1 |
| Sod1 | Mm01700393-g1 |
| Tnf | Mm00443258-m1 |
| Tnfrsf1b | Mm00441875-m1 |
| Wnt1 | Mm01300555-g1 |
| Th | Mm 00447546-m1 |
