# Supplementary material for: Boosting Antioxidant Self-defenses by Grafting Astrocytes Rejuvenates the Aged Microenvironment and Mitigates Nigrostriatal Toxicity in Parkinsonian Brain via an Nrf2-Driven Wnt/β-Catenin Prosurvival Axis
Source: Front Aging Neurosci. 2020 Mar 12;12:24. doi: 10.3389/fnagi.2020.00024 (PMC7081734; doi:10.3389/fnagi.2020.00024)
Supplement: Supplementary file 3 [file Table_3.DOCX]

**Supplementary Table 3. Classification of microglia activation in uninjured and after MPTP treatment in the absence or the presence of t-VM-As grafts.**

_________________________________________________________________________

**Stages of microglia activation Saline/PBS MPTP/PBS MPTP/t-VM-AS**

**Cell percentages/treatment group**

**________________________________________________________________________________**

**Stage 1**: Resting microglia. Rod shaped

Soma,fine and ramified processes. ≥50±10 ≤9 ± 3 ≥48± 12

**Stage 2**: Activated ramified microglia,

with elongated shaped body ≥ 35 ± 6 ≤20 ± 8 ≥40± 10

long and thicker processes.

**Stage 3**: Ameboid microglia. Round shaped ≤10 ± 2 ≥ 40± 12** ≤8± 2

body with short, thick/stout processes.

**Stage 4**: Phagocytic cells. Round shaped cells ≤5± 2 ≥ 30± 9** ≤5± 2

with vacuolated cytoplasm, no processes.

___________________________________________________________________________

Stages of microglia activation were studied according to Kreutzberg (1999) in midbrain sections at the level of the SNpc in saline/PBS, MPTP/PBS and MPTP/t-VM-As grafted mice during the course of the experimental period, IBA1+ cells were analyzed by confocal laser microscopic analyses in 8-10 randomly selected fields/section on both sides as described in the Method section. Results obtained at 4 wpt are shown. Data are expressed as changes in percentages (%) (mean±SEM, in n = 5 mice/experimental group). In MPTP/PBS, a greater proportion of stage 3 and 4 microglia were observed vs saline/PBS and MPTP/t-VM-As. By contrast, MPTP/t-VM-As, more stage 1 and 2 were counted. ** *p*≤ 0.01, vs saline/PBS and MPTP/t-VM-As, by ANOVA.
