# Supplementary figures and images for: Boosting Antioxidant Self-defenses by Grafting Astrocytes Rejuvenates the Aged Microenvironment and Mitigates Nigrostriatal Toxicity in Parkinsonian Brain via an Nrf2-Driven Wnt/β-Catenin Prosurvival Axis
Source: Front Aging Neurosci. 2020 Mar 12;12:24. doi: 10.3389/fnagi.2020.00024 (PMC7081734; doi:10.3389/fnagi.2020.00024)

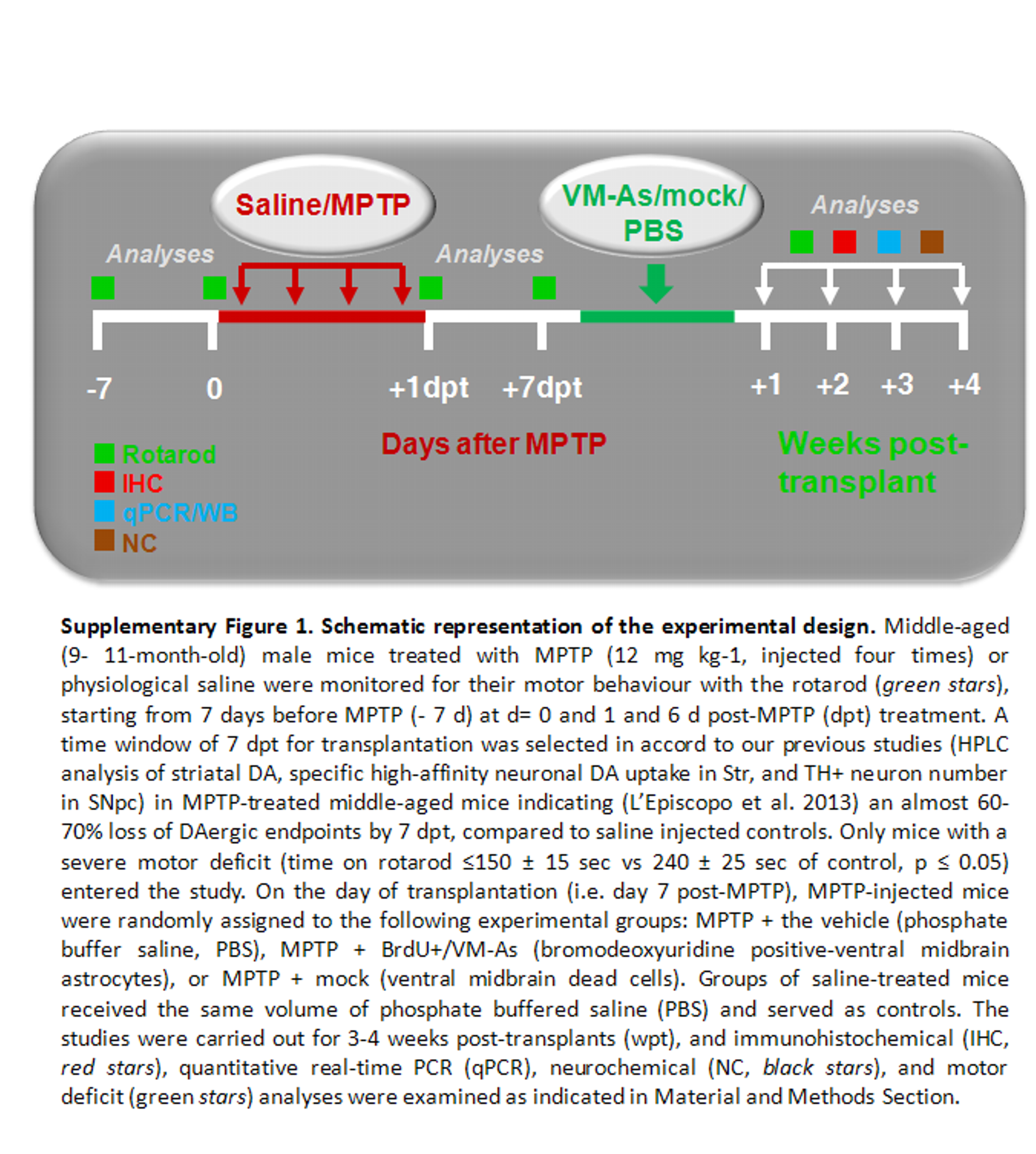

Supplement: Supplementary file 4 [file Image_1.TIF]
